# Phenotype–genotype network construction and characterization: a case study of cardiovascular diseases and associated non-coding RNAs

**DOI:** 10.1093/database/baz147

**Published:** 2020-01-15

**Authors:** Rongrong Wu, Yuxin Lin, Xingyun Liu, Chaoying Zhan, Hongxin He, Manhong Shi, Zhi Jiang, Bairong Shen

**Affiliations:** 1 Center for Systems Biology, Soochow University, No. 199 Renai Road, Suzhou, Jiangsu 215123, China; 2 Institutes for Systems Genetics, West China Hospital, Sichuan University, No. 17 Gaopeng Avenue, Ji Tai’an Center, Chengdu, Sichuan 610041, China; 3 College of Information and Network Engineering, Anhui Science and Technology University, No. 9 Donghua Road, Fengyang, Anhui 233100, China; 4 Department of Biochemistry and Molecular Biology, School of Medicine, Soochow University, No. 199 Renai Road, Suzhou, Jiangsu 215123, China

## Abstract

The phenotype–genotype relationship is a key for personalized and precision medicine for complex diseases. To unravel the complexity of the clinical phenotype–genotype network, we used cardiovascular diseases (CVDs) and associated non-coding RNAs (ncRNAs) (i.e*.* miRNAs, long ncRNAs, etc.) as the case for the study of CVDs at a systems or network level. We first integrated a database of CVDs and ncRNAs (CVDncR, http://sysbio.org.cn/cvdncr/) to construct CVD–ncRNA networks and annotate their clinical associations. To characterize the networks, we then separated the miRNAs into two groups, i.e*.* universal miRNAs associated with at least two types of CVDs and specific miRNAs related only to one type of CVD. Our analyses indicated two interesting patterns in these CVD–ncRNA networks. First, scale-free features were present within both CVD–miRNA and CVD–lncRNA networks; second, universal miRNAs were more likely to be CVDs biomarkers. These results were confirmed by computational functional analyses. The findings offer theoretical guidance for decoding CVD–ncRNA associations and will facilitate the screening of CVD ncRNA biomarkers.

**Database URL**: http://sysbio.org.cn/cvdncr/

## Introduction

In the era of personalized and precision medicine, the relationship between clinical phenotypes and their genotypes are central for precisely classifying diseases. Until now, there are few studies of phenotype–genotype networks (1,2). To unravel the complexity of clinical phenotype–genotype relationships and to identify patterns in complex networks, we used cardiovascular diseases (CVDs) and associated non-coding RNAs (ncRNAs) to construct and characterize a phenotype–genotype network, whereby different CVD subtypes were treated as clinical phenotypes and their associated ncRNAs as genotypes.

Establishment of high-throughput transcriptome analysis of mammalian genomes and its wide application have facilitated the identification of many ncRNAs (3). The coding exons in protein-coding genes account for only 1.5% of the genome (4), and as much as 50% of the transcriptome has no protein-coding potential (5). Nonetheless, increasing evidence indicates that certain ncRNAs have critical pathophysiological functions in diseases and in the regulation of biological processes, such as differentiation, development and post-transcriptional regulation of gene expression (6,7). ncRNAs, including microRNAs (miRNAs), long ncRNAs (lncRNAs), circular RNA (circRNAs) and other types (4), are RNA molecules that are transcribed but not translated into a protein, though they do comprise an important class of regulatory molecules responsible for the fine tuning of gene expression (5) at the RNA level. Indeed, the importance of ncRNAs has attracted interest in the field of bioscience, and these molecules have been investigated in many fundamental biological and pathophysiological studies (8). Furthermore, ncRNAs regulate various genes related to development of the mammalian heart, with known associations with human CVDs (9,10).

Among the various types of ncRNAs, miRNAs have been most widely studied in recent years, and these endogenous, highly conserved small RNA molecules of ~22 nucleotides are widespread in eukaryotes (11). In general, miRNAs bind to a complementary section of cognate mRNAs and exert negative regulatory effects on protein synthesis by inhibiting translation or by promoting degradation of the mRNA. According to the definition of the National Institutes of Health, miRNAs can also serve as biomarkers to predict changes in biological processes or responses to therapies (12–15). There have also been various reports of miRNA involvement in CVDs. For instance, the level of miR-let-7i is decreased in the circulating leukocytes of patients with acute ischemic stroke, regulating the inflammatory response post-stroke (16). Additionally, miR-33 participates in cardiac remodeling, where it helps to maintain the cholesterol level and adaptive fibrotic responses in patients with heart failure (17). In acute myocardial infarction patients, miR-378 acts as a key regulator of the proangiogenic capacity of CD34^+^ progenitor cells, with stimulatory effects on endothelial cells by eliciting an innovative endogenous repair mechanism (18). Moreover, miR-2909 regulates genes associated with inflammation and immunity, contributing to the initiation and development of pathophysiological processes in coronary heart disease (19,20).

In addition to miRNAs, the roles of some other ncRNAs have been determined, such as lncRNAs and circRNAs, and the number of related studies is growing correspondingly (21). In particular, Rhabdomyosarcoma 2-associated transcript is involved in the pathogenesis of ischemic brain injury, and it inhibits middle cerebral artery occlusion-induced ischemic brain injury during treatment of ischemic stroke patients (22). The lncRNA TRDN-AS (denoting “triadin antisense”) facilitates a balance between cardiac and skeletal isoforms of triadin, and it has been proposed for use in a candidate treatment strategy for heart failure (23). As high stability is a common feature of circRNAs, they may be employed as biomarkers for CVDs. Furthermore, circular antisense ncRNAs in the INK4 locus (cANRIL) regulates ribosome RNA biogenesis to protect against atherosclerosis (24). These types of ncRNAs are the focus of the present study.

**Figure 1:**
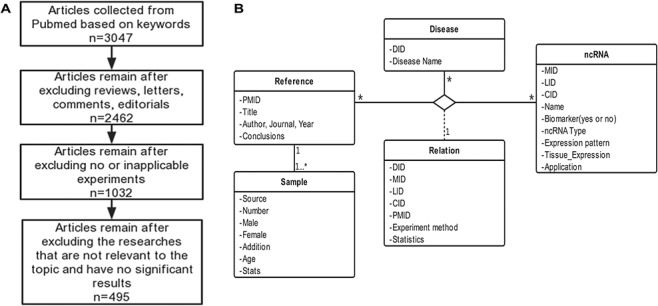
Schematic workflow of CVDncR. (A) The workflow of data filtering. (B) The E-R model of the CVDncR database.

CVDs are major causes of morbidity and mortality, imposing immense health and economic burdens worldwide (25). The global number of deaths due to CVDs is as high as 15 million each year, and morbidity and mortality rates are increasing (26). Although a wide range of therapeutic and preventative approaches have proven effective in CVDs, both developed and developing countries still face serious challenges (27). Due to the increasing number of patients with CVDs, current clinical work is focused on understanding their pathogenesis and preventing their occurrence and recurrence as well as curing refractory heart disease. Recent studies have shown that ncRNAs can be employed as latent therapeutic targets for various CVDs and as diagnostic or prognostic markers (28–31). Although the number of ncRNAs research is increasing gradually, the underlying mechanisms are still largely unknown. Interactions among ncRNAs contribute to the occurrence and development of CVDs. For example, lncRNAs have been reported as the miRNA sponge, which could produce negative regulation to the miRNA, or directly target on the miRNA to regulate gene expression. Thus, constructing and characterizing miRNA-mediated ternary network would help the discovery of potential pathogenesis for systems-level CVD understanding.

Based on laboratory research and a large amount of fundamental data, ncRNAs are known to have strong regulatory effects on CVDs, but a database describing the relationships between ncRNAs and CVDs to allow the systematic study of ncRNA-CVDs network is lacking. In the present study, we developed a manually curated and high-quality database to manage large amounts of information regarding relationships between ncRNAs and CVDs and allows researchers to conduct data searches using this resource platform, statistical and bioinformatics analyses also can be conducted based on it. Using this database, we constructed a network of interactions between CVDs and ncRNAs, explored associations, correlations and network distributions, and determined how ncRNAs can affect diseases via genes and signaling pathways. These issues are particularly important for research into CVDs and the development of precision medicine. By using 23 different CVD subtypes as clinical phenotypes and their associated ncRNAs as genotypes, we constructed a phenotype–genotype network to understand their relationships and to identify general network patterns at the systems biology level.

## Results

### Database entities and contents

CVDncR (http://sysbio.org.cn/cvdncr/) is a database of manually collected data representing ncRNAs related to CVDs. The database contains content related to miRNAs, lncRNAs, and circRNAs for 23 different CVDs. The workflow of the data-filtering process for the CVDncR database is shown in Figure 1A. At present, the database contains 426 miRNA sentries, 99 lncRNA entries and 24 circRNA entries from 495 published articles (Table 1). The 23 diseases included in this database are listed in Figure 3. The details of the database are illustrated in the E-R model depicted in Figure 1B, containing all detailed information of each item in the CVDncR database.

**Figure 2:**
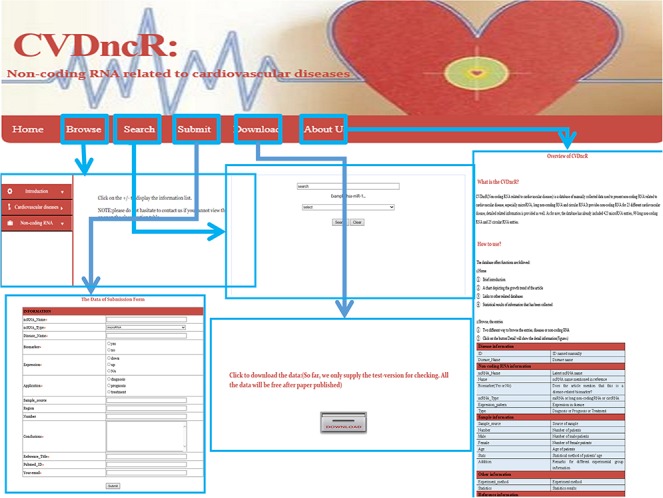
The implementation and overview of the CVDncR database.

**Table 1:** Article screening results

Number	miRNA	lncRNA	circRNA
Articles originally searched	2848	144	55
Articles ultimately contained	433	48	14
Ultimately entries	426	99	24

### User interface

The CVDncR database provides a user-friendly interface for various types of data through six pages (Figure 2). The functions of the interfaces are summarized below.

1. Home Page. This page has four components: (i) a brief introduction to the purpose of the database; (ii) a chart depicting the growth trends in articles; (iii) links to other related databases, such as miRBase and (iv) statistical results for the collected information.
2. Browse Page. The “Browse” interface is the key component of the database library. To browse all ncRNAs and their relationships with CVDs, users can click on the “Browse” menu on the website. For convenience, the information accessed via this page is presented in two main ways, by either browsing the “CVDs” list or the “ncRNAs” list. The catalog of CVDs includes 23 different diseases. If the user clicks on a specific CVD button, all recorded relevant data will be shown in the window. The detailed information for each item can be accessed by clicking the “more” button at the end of the entry.
3. Submit Page. If there has been a new discovery or the CVDncR database lacks specific data, users can submit novel validated items or send an email. The new record will be listed in the database after the submission has been approved.
4. Search Page. CVDncR allows direct searching via the “Search” page. The user can input a specific ncRNA name or CVD name, and the corresponding results will be provided in a new window.
5. Download Page. CVDncR is freely available for use and download. The “Download” page allows users to acquire all curated entries related to CVDs.
6. About Us Page. The “Help” page provides an overview and a tutorial for CVDncR, including “what’s CVDncR” and “How to use.”

### Comparison with existing databases

Thus far, many well-constructed ncRNA databases for disease research have been reported (32), such as HMDD (33), LncRNADisease V2.0 (34), HDncRNA (35), miR2Disease (36), LncRNome (37), lncRNAdb V2.0 (38), NONCODE V5.0 (39), Lnc2Cancer V2.0 (40), CSCD (41), circRNADb (42), deepBase v2.0 (43) and circBase (44). As illustrated in Table 2, our database (CVDncR) has the following advantages.

1. As a database specific for CVDs, CVDncR records ncRNA information for more than 20 CVD types;
2. CVDncR is a comprehensive database of three types of ncRNAs, i.e*.* miRNAs, lncRNAs and circRNAs, associated with CVDs;
3. The annotations in our database contain specific information for future translational applications, such as patient sample information and biomarker information for diagnosis, treatment and prognosis, etc.

**Table 2:** Comparison with other databases

Databases/function	miRNA	lncRNA	circRNA	CVD subtypes	Expression	Tissue_expression	Application (D, P, T)	Sample information	Biomarker
CVDncR	**•**	**•**	**•**	**•**	**•**	**•**	**•**	**•**	**•**
HMDD	**•**	**◯**	**◯**	**•**	**◯**	**◯**	**◯**	**◯**	**◯**
miR2Disease	**•**	**◯**	**◯**	**•**	**•**	**◯**	**◯**	**◯**	**◯**
HDncRNA	**•**	**•**	**•**	**•**	**•**	**◯**	**◯**	**◯**	**◯**
LncRNADisease V2.0	**◯**	**•**	**•**	**•**	**◯**	**◯**	**◯**	**◯**	**◯**
LncRNome	**◯**	**•**	**◯**	**◯**	**◯**	**◯**	**◯**	**◯**	**◯**
lncRNAdb V2.0	**◯**	**•**	**◯**	**◯**	**◯**	**◯**	**◯**	**◯**	**◯**
NONCODE V5.0	**◯**	**•**	**◯**	**◯**	**◯**	**◯**	**◯**	**◯**	**◯**
Lnc2Cancer V2.0	**◯**	**•**	**◯**	**◯**	**•**	**◯**	**•**	**◯**	**◯**
CSCD	**◯**	**◯**	**•**	**◯**	**◯**	**◯**	**◯**	**◯**	**◯**
circRNADb	**◯**	**◯**	**•**	**◯**	**◯**	**◯**	**◯**	**◯**	**◯**
deepBase V2.0	**◯**	**◯**	**•**	**◯**	**◯**	**◯**	**◯**	**◯**	**◯**
circBase	**◯**	**◯**	**•**	**◯**	**◯**	**◯**	**◯**	**◯**	**◯**

Abbreviation: D, P, T: diagnosis, prognosis, treatment.

### Deciphering the phenotype-genotype (CVD–ncRNA) network

**Figure 3:**
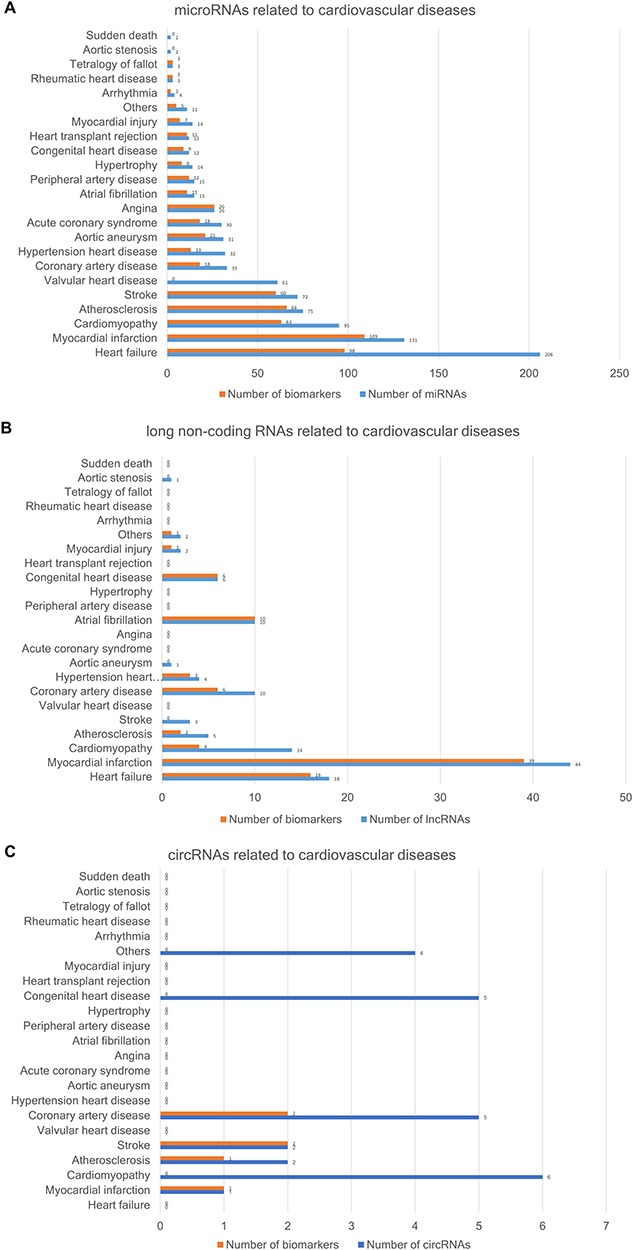
The number of ncRNAs related to CVDs. (A) The number of miRNAs. (B) The number of lncRNAs. (C) The number of circRNAs.

The CVDncR database contains 426 miRNAs, 99 lncRNAs and 24 circRNAs associated with 23 different CVDs. The number of ncRNAs associated with each CVD type is illustrated in Figure 3. Figure 3A shows the number of miRNAs or biomarkers related to different CVDs. It is clear that heart failure ranks first with the highest number of related miRNAs. As shown in Figure 3B, myocardial infarction has the most associated lncRNAs. The result shown in Figure 3C indicated that most of the reported circRNAs are associated with cardiomyopathy.

**Figure 4:**
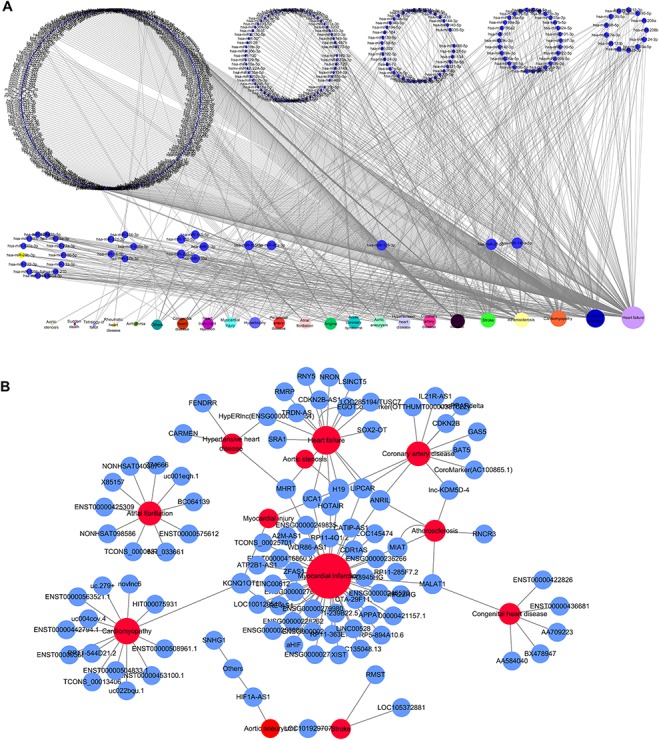
Network distribution of ncRNAs related to CVDs. (A) Overview of the miRNA–CVD network; the bottom row shows the diseases, and the size of the circles is proportional to the number of associated miRNAs. (B) Overview of the lncRNA–CVD network; red circles represent diseases, blue circles are lncRNAs and the size of the circles is proportional to the number of associated lncRNAs.

**Figure 5:**
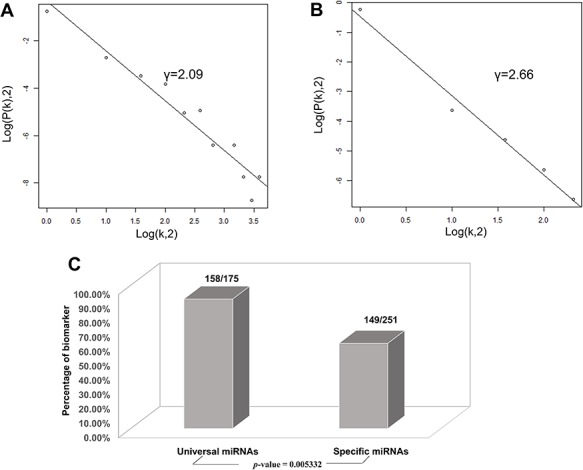
Correlations and distribution of miRNAs and lncRNAs related to CVDs. The *x*-coordinate *K* is the number of related CVDs, and the *y*-coordinate is the proportion of ncRNAs (miRNAs or lncRNAs). (A) Scale-free feature of lncRNAs related to CVDs. (B) Scale-free feature of lncRNAs related to CVDs. (C) Percentage of biomarkers in universal miRNAs and specific miRNAs.

Based on the database, we found that few miRNAs are associated with more than a dozen diseases; examples are miR-146a-5p and miR-21-5p, which are associated with up to 12 types of CVDs. In addition to these, a large proportion of miRNAs are associated with a few diseases, such as miR-126-3p and miR-155-5p*.* Conversely, some other miRNAs, such as let-7b-3p and miR-105-5p, are associated with only one CVD. Regarding lncRNAs, H19 is associated with five types of CVDs, though the majority are only associated with one disease. As there are presently very few studies reported on CVD-associated circRNAs, we did not perform statistical analyses. Figure 4A and 4B respectively shows the correlation among the proportions and distribution patterns between CVDs, miRNAs, and lncRNAs. In Figure 4A, the blue dots represent miRNAs, and the other dots represent various CVDs. The larger the cardiovascular dot, the more associated miRNAs the disease has, and the larger the miRNA dot, the more CVDs associated with it. Similarly, in Figure 4B circles represent diseases, blue circles are lncRNAs and the size of the circles is proportional to the number of associated lncRNAs. The figures are drawn using CytoScape software tool. Barabasi and Albert first proposed the scale-free model in 1991 (45), in which the distribution *P*(*k*) of degrees of nodes (*k*) in the networks follows a power law, $P$($k$)~${k}^{-\gamma }$, where the scaling parameter *γ* typically lies in the range 2 < *γ* < 3 (46). Some nodes called “hubs” have many more connections than do others, and they determine most of the functions and features of the entire network. Interestingly, we identified this pattern in the CVD–ncRNA (phenotype–genotype) network, as presented in Figure 5A and B. The parameter *γ* was determined to be 2.09 for CVD–miRNA and 2.66 for CVD–lncRNA association distributions. All calculations have been carried out under R version 3.0.2.

We then investigated whether miRNAs associated with more CVDs are more potent biological regulators. The miRNAs were classified into two categories, i.e*.* universal miRNAs associated with at least two types of CVDs and specific miRNAs related only to one type of CVD. Based on their classifications and interactions with target genes, we found the following:

1. Universal miRNAs comprising 175 miRNAs corresponding to 7479 genes, including 24 937 miRNA–mRNA interactions;
2. Specific miRNAs comprising 251 miRNAs corresponding to 5445 genes, including 11 444 miRNA–mRNA interactions (see Supplementary File 1).

The number of universal miRNAs was much lower than that of specific miRNAs. The target genes and miRNA–mRNA interactions indicated that universal miRNAs regulate many more genes than do specific miRNAs.

### Gene ontology annotation and pathway analysis

Database for annotation, visualization and integrated discovery (DAVID) was applied for gene ontology (GO) annotation at three levels: molecular function, biological process and cellular component (47). There were 1102 chart records for GOTERM_BP_FAT among the universal miRNAs, 962 for specific miRNAs. There were 228 chart records for GOTERM_MF_FAT among the universal miRNAs, 218 for specific miRNAs. The GO analysis results indicated that there were fewer universal miRNAs than specific miRNAs, with greater enrichment at the biological process and molecular function levels.

Pathway analysis was based on Ingenuity Pathway Analysis (IPA, QIAGEN). Signaling pathways enriched by targets of the two types of miRNA with *P*-values < 0.01 were collected; we observed 27 and 42 enriched pathways for specific and universal miRNAs, respectively, with 18 of them overlapping. Greater participation in more enriched pathways indicates contribution to more biological processes, indirectly explaining why specific miRNAs are associated with only one type of CVD, whereas universal miRNAs are associated with multiple CVDs simultaneously, the corresponding IPA enrichment results are listed in Supplementary File 2. The top 10 significantly enriched pathways screened by IPA are shown in Table 3**,** including *P*-value and ratio of enriched genes in each pathway.

**Table 3:** Top 10 significantly enriched pathways for the two miRNA sub-types

No.	Ingenuity canonical pathways	*P*-value	Adjusted *P*-value (FDR)	Ratio of enriched genes in the pathway	Description	Reference (PMID)
				Specific miRNAs		
1	Small cell lung cancer signaling	5.50E-04	7.20E-02	0.824	N/A	N/A
2	Production of nitric oxide and reactive oxygen species in macrophages	5.50E-04	7.20E-02	0.824	Overproduction of nitric oxide by inducible nitric oxide synthase in macrophages of atherosclerosis has a negative effect	16 335 796
3	Pancreatic adenocarcinoma signaling	7.76E-04	7.20E-02	0.789	Treated with gemcitabine in pancreatic adenocarcinoma with cardiovascular dysfunction may trigger systemic syndrome	15 630 853
4	B cell receptor signaling	7.76E-04	7.20E-02	0.789	Downregulation of B cell receptor is involved in the immune system suppression stimulated by inflammation in CVDs	27 213 032
5	UVB-induced MAPK signaling	8.91E-04	7.20E-02	0.909	Long non-coding RNA MALAT1 might play a role in UVB-induced photoaging via regulation of the MAPK pathway in CVDs	28 487 970
6	Tec kinase signaling	2.34E-03	1.36E-01	0.8	T-cell kinase is relevant to T cell development and regulates inflammatory responses in myocarditis aroused by coxsackievirus B3 infection	24 462 896
7	Role of tissue factor in cancer	3.09E-03	1.36E-01	0.765	The level of a tissue factor pathway inhibitor is elevated in patients with varieties of CVDs	16 355 095
8	UVA-induced MAPK signaling	3.39E-03	1.36E-01	0.833	UVA inhibits adipogenic differentiation and migration by mediating MAPK in heart disease	20 693 579
9	Sumoylation pathway	3.39E-03	1.36E-01	0.833	Sumoylation promotes apoptosis via improving conjugation in cardiomyopathy and heart failure	25 857 621
10	HGF signaling	3.72E-03	1.36E-01	0.737	Upregulation of HGF is associated with its utilization as a biomarker for several CVDs	29 321 335, 28 572 400, 27 342 109
				Universal miRNAs		
1	Molecular mechanisms of cancer	6.61E-05	2.63E-02	0.867	N/A	N/A
2	Role of tissue factor in cancer	1.62E-04	2.63E-02	1	The level of tissue factor pathway inhibitor is elevated in patients with varieties of CVDs	16 355 095
3	RAR activation	3.09E-04	2.63E-02	0.952	N/A	N/A
4	ILK signaling	3.09E-04	2.63E-02	0.952	ILK disorder cell migration is mediated by SDF-1 in myocardial hypertrophy and idiopathic dilated cardiomyopathy patients	24 075 768, 20 493 167
5	p53 signaling	4.68E-04	2.63E-02	1	p53 is crucial for the cell cycle and migration in CVD patients	24 700 313, 21 630 092
6	Estrogen-dependent breast cancer signaling	4.68E-04	2.63E-02	1	N/A	N/A
7	Aryl hydrocarbon receptor signaling	5.01E-04	2.63E-02	0.95	AHR signaling pathway is involved in the process of hypertensive patients	27 977 510
8	IGF-1 signaling	5.01E-04	2.63E-02	0.95	IGF-1 is an aging indicator that participates in promoting the aging process in patients diagnosed with CVDs	29 393 358
9	Colorectal cancer metastasis signaling	5.50E-04	2.63E-02	0.861	N/A	N/A
10	Germ cell-sertoli cell junction signaling	8.13E-04	2.92E-02	0.947	N/A	N/A

Abbreviation: N/A: not available.

We also evaluated the relevance of the identified pathways by searching PubMed for published studies describing the roles of network objects in pathways, as provided in Table 3. We verified 13 of the 19 pathways based on relevant studies, and thus our pathway enrichment results confirmed that the target genes corresponding to the miRNAs collected in the database have strong relationships with CVDs. Overall, we verified the accuracy and importance of the miRNAs collected in the database according to their participation in many biological processes related to the development of CVDs.

Additionally, verification based on the DEG database indicated that 3146 essential genes among the 5445 genes (i.e*.* 57.8%) are targets of specific miRNAs and that 4045 essential genes among the 7479 genes (i.e*.* 54.1%) are targets of universal miRNAs. Therefore, many of the miRNAs collected in the database and their target genes are essential for the development of CVDs in organisms, reflecting the importance of these miRNAs.

Finally, we conducted statistical analyses of biomarkers: among the 426 miRNAs, 99 lncRNAs and 24 circRNAs, 306, 77 and 6 were verified as biomarkers of CVDs in related studies, respectively. Most studies have focused on biomarkers for myocardial infarction, followed by heart failure. We found 90.3 and 59.4% of the universal miRNAs and specific miRNAs, respectively, to be biomarkers (*P*-value = 0.005332), as illustrated in Figure 5C. Thus, miRNAs associated with various types of CVDs are more likely to serve as biomarkers**.**

### Literature-guided ternary network analysis

Increasing evidence supports the involvement of ncRNAs in many pathological cardiovascular conditions (48). Although some studies have assessed their functions in cell-to-cell communication in the surrounding environment, the consequences and biological effects of these disease-associated ncRNAs remain unclear (49), and the underlying mechanisms are largely unknown. We analyzed how the ternary networks of different ncRNAs and diseases might influence disease progression by constructing networks comprising miRNAs, lncRNAs and circRNAs. The database contains 14 pairs of lncRNAs/miRNAs and three pairs of circRNAs/miRNAs associated with specific CVDs, which were provided in Table 4. Furthermore, we evaluated these lncRNA–miRNA–mRNA and circRNA–miRNA–mRNA networks to investigate how lncRNAs or circRNAs affect regulatory relationships between miRNAs and CVDs.

**Table 4:** Lists of ternary networks

	miRNA	mRNA
lncRNA		
MIAT	miR-181b	STAT3
SNHG1	miR-195	BCL2L2
Kcnq1ot1	miR-214-3p	caspase-1
APPAT	miR-647	NA
MALAT1	miR-214	XBP1
XIST	miR-130a-3p	PDE4D
HOTAIR	miR-1	NA
UCA1	miR-1	BCL2/HSP60
RNCR3	miR-185-5p	KLF2
H19	miR-103	FADD
H19	miR-107	FADD
novlnc6	miR-133a	BMP10/Nxk2.5
novlnc6	miR-2499	BMP10/Nxk2.5
novlnc6	miR-30c	BMP10/Nxk2.5
circRNA		
circRNA_007878	let-7e	NA
circDLGAP4	miR-143	HECTD1
circR-284	miR-221	NA

#### The lncRNA–miRNA–mRNA networks

*LncRNAs function as competitive endogenous RNAs.* It has been suggested that naturally occurring ncRNA transcripts can act as endogenous miRNA sponges or competing endogenous RNAs (ceRNAs) (50). Indeed, increasing experimental evidence supports the hypothesis that lncRNAs and circRNAs may act as miRNA sponges to sequester miRNAs (51), thereby enhancing the expression and function of target mRNAs and negatively regulating miRNAs.

Analysis of serum from atherosclerosis patients has shown upregulation of myocardial infarction-associated transcript (MIAT) expression and inverse regulation of miR-181b expression who were revealed that block cell growth, induce cell cycle arrest, impede proliferation and promoting apoptosis. Furthermore, MIAT greatly enhances signal transducer and activator of transcription 3 (STAT3) expression by acting as a molecular sponge to sequester miR-181b, promoting the development and progression of atherosclerosis via the miR-181b/STAT3 regulatory pathway, however, this effect is counteracted by miR-181b (52) overexpression. Zhang *et al*. (53) also found that small nucleolar RNA host gene 1 (SNHG1) functions as a ceRNA to regulate expression of miR-195 in human cardiomyocytes, with overexpression of SNHG1 attenuating the effects of hydrogen peroxide treatment to significantly increase the rate of apoptosis and decrease cell viability. As miR-195 reportedly negatively regulates expression of BCL-2-like protein 2 (BCL2L2), modifying the level of SNHG1 might constitute a novel strategy for treating cardiomyocyte apoptosis-related heart diseases.

*LncRNAs directly target miRNAs.* Major downregulation of atherosclerotic plaque pathogenesis-associated transcript (APPAT) has been found in coronary artery samples from patients with severe stenosis, and the level of circulating APPAT correlates negatively with that of circulating miR-647, which has been identified as a candidate target of APPAT. Thus, dynamic changes in the level of APPAT might indicate disease stage in clinical diagnostic applications (54). Additionally, by directly regulating miR-214, the MALAT1 rs619586 G allele is associated with a higher risk of pulmonary arterial hypertension (55).

In post-myocardial infarction cells, it has been confirmed that X-inactive specific transcript RNA (XIST) can negatively mediate miR-130a-3p expression, and miR-130a-3p represses expression of PDE4D. Accordingly, XIST may suppress the proliferation of post-myocardial infarction cells and promote apoptosis by targeting miR-130a-3p and PDE4D (56) in a straight line.

#### The circRNA–miRNA–mRNA networks

CircRNAs may contribute to the occurrence of CVDs by acting as miRNA sponges or by binding to proteins, with possible important roles in heart development. Moreover, bioinformatics analyses of molecular functions have shown that circRNAs may have roles similar to those of their host genes and they may function via protein binding (57).

In addition, circDLGAP4 levels are significantly decreased and miR-143 upregulated in the plasma of acute ischemic stroke patients. circDLGAP4 functions as an endogenous miR-143 sponge to inhibit the activity of miR-143, resulting in increasing expression of HECT domain E3 51 ubiquitin protein ligase 1 (HECTD1), a target of miR-143. Restoring circDLGAP4 was able to decrease areas of infarct and diminish neuronal deficits, and it may be a unique therapeutic strategy for emergency ischemic injury treatment (58).

It has also been validated that the circR-284 to miR-221 ratio is significantly increased in the serum of urgent symptomatic carotid disease patients after plaque rupture; circR-284 may act as a miR-221 sponge to restrain its activity, where it may have high sensitivity and specificity as a diagnostic biomarker for carotid plaque rupture and stroke (59).

#### Cluster analysis of ncRNA tissue expression

**Figure 6:**
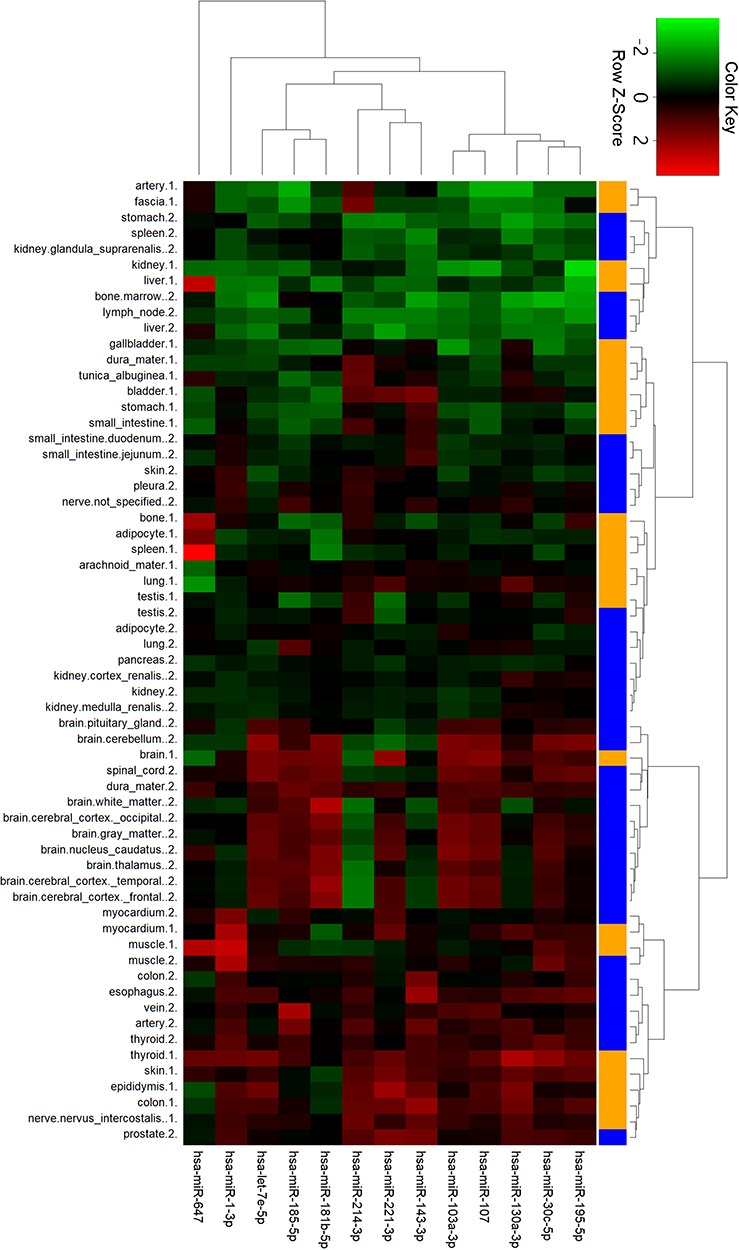
Cluster analysis of miRNA tissue expression profiles in ternary network. The orange and blue codes on the right side represent the tissue samples from two male bodies, i.e., a patient with myeloma and a male under a natural death.

To gain further insights into tissue expression of specific miRNAs, we performed clustering analysis with the 13 miRNAs in ternary networks (Figure 6). Interestingly, two groups of miRNAs with differential expression patterns were shown. In detail, miRNAs in the first group with stomach, kidney, liver, spleen and bone tissues were highly expressed. On the contrary, miRNAs were low expressed in the cluster of myocardial, muscle, colon and thyroid tissues. Four miRNAs, i.e. miR-1-3p, miR-647, miR-181b-5p and miR-143-3p, were also detected to be significantly expressed in myocardial and artery tissues.

## Discussion

Despite the therapeutic benefits of numerous treatment options to combat CVDs, including angiotensin-converting enzyme inhibitors, statins and other drugs, their incidence continues to increase, and there is a need for new therapeutic approaches (60). It is essential to study ncRNAs related to CVDs (61) because of their underlying roles in physiological and pathological processes (49). In fact, research into the relationships between ncRNAs and CVDs has been increasing for nearly 10 years, and the number of studies in PubMed is growing exponentially. Numerous ncRNAs may be potential targets for pharmacological therapy, but more studies are needed to determine whether they will ultimately have clinical application (62).

In the present study, we developed the CVDncR database by systematically collecting and storing important biomedical information regarding the relationships between ncRNAs and CVDs. Compared with existing databases, CVDncR records miRNA, lncRNA and circRNA information for 23 CVD types and had a broad range of contents for data annotation. Moreover, the CVDncR database contains experimentally validated ncRNAs in humans, as well as clinical specific information such as biomarkers for diagnosis, treatment and prognosis, which can be helpful for systems biology analyses of ncRNA biomarkers and clinical applications.

The focus of the present study is on phenotype-genotype relationships, i.e*.* the relationship between phenotype and genotype, and CVD–ncRNA networks. We conducted statistical analyses based on our database and constructed binary networks according to interactions between ncRNAs and CVDs. We found scale-free distributions for both miRNAs and lncRNAs with CVDs, where 1.4% of the miRNAs were associated with 82.6% of the CVDs and a small number of ncRNAs with most of the diseases. We identified universal miRNAs that are more likely to serve as biomarkers of diseases, with stronger regulating power than specific RNAs. Pathway analysis showed that most of the 10 enriched signaling pathways for both specific and universal miRNAs are supported by published studies, with smaller *P*-values for enriched pathways associated with universal miRNAs, indicating that they are more strongly associated with CVDs. To decrease false positive results and increase the accuracy of pathway enrichment analysis, only four experimentally validated databases, i.e*.* miRTarBase, miRecords, StarBase and TarBase, were selected in this study to determine the targets of miRNAs. Nonetheless, some computational tools such as mirDIP (63,64) are also powerful for miRNA-target identification, and we will integrate computationally predicted data in our future work.

It is worth mentioning that the significantly enriched pathways obtained are mainly cancer-related pathways and immune-related pathways. Studies have shown that there is extensive overlap in terms of risk factors and disease prevention characteristics related to CVD and cancer, suggesting that these diverse diseases share common biological mechanisms and mutual influences and that sociodemographic factors are also associated with the risk of CVDs (65,66). One case-controlled study conducted among women with breast cancer in Sweden and Denmark who underwent radiation therapy showed that the rates of major coronary events had linear associations with the radiation dose, and an increased risk of CVD in women who survive breast cancer has also been demonstrated (67,68). CVD is the leading cause of death in chronic kidney disease patients, and Bae *et al*. (69) demonstrated that circulating tumor necrosis factor receptor can be used as an indicator for the occurrence of CVD in chronic kidney disease patients, and patients with non-small cell lung cancer exhibit adverse effects of treatment, potentially leading to congestive heart failure (70). Moreover, many experimental and clinical studies have shown that the pathology of autoimmune inflammation plays an important role in CVDs (71). It is known that the immune system is a powerful contributor to the development of CVD, where it can be activated and produce an abnormal immune response, with the abnormal expression of inflammatory cytokines (72). In acute coronary syndromes, T cells stimulate IFN-gamma, which leads to specific immune responses (73). T cells in the atherosclerotic lesions of patients with coronary artery disease also exert a protective action via the secretion of antibodies, which results in inflammation (74). Nonetheless, further explorations of the effects of immunotherapy are required.

GO analysis indicated the universal miRNAs to be associated with more biological processes and molecular functions than the specific miRNAs. The lncRNAs comprising H19, ANRIL and KCNQ1OT1 exhibit positive and significant correlations with CVDs. According to human experiments, the circRNA referred to as myocardial infarction-associated circular RNA is associated with an increased risk of myocardial infarction (30). Although studies of miRNAs and lncRNAs are maturing, investigation of circRNAs is still in its infancy, and more efforts are needed to identify the roles of ncRNAs in cardiovascular pathology as well as their possible applications.

Studies of the independent regulatory effects of different ncRNAs have mainly been excessively focused, and further research into their joint effects on CVDs is needed. In addition, in the pathway enrichment, the corresponding *P*-value results were significant, however, the adjusted *P*-values calculated by FDR, BH and Bonferroni methods lacked enough significance. More validation studies should be performed to decode the association between the miRNAs and CVD pathogenesis. According to previous studies, lncRNAs and circRNAs act as ceRNAs for miRNAs in CVDs, and lncRNAs can control gene expression by acting as sponges for miRNA in their regulatory relationships. Therefore, we constructed ternary networks by combining genes and signaling pathways to understand the key features of ncRNAs in these networks.

## Conclusions

In this study, we developed the CVDncR database based on reported miRNAs, lncRNAs, and circRNAs and analyzed CVD–ncRNA networks. The results indicate two interesting patterns. First, scale-free features are present within both CVD–miRNA and CVD–lncRNA networks. Second, universal miRNAs were found to be more likely to act as CVD biomarkers. These findings were confirmed by computational functional analyses and therefore provide theoretical guidance for the decoding of CVD–ncRNA associations and facilitate the screening of CVD ncRNA biomarkers clinically.

## Materials and methods

### Data collection

Details of various types of CVDs were collected to construct the CVDncR (ncRNAs related to CVDs) database. To ensure that the data were comprehensive, we searched for information regarding CVDs in ICD-10, version 2016, where number I05-I79 was included in the subclass “Diseases of circulatory system” for CVDs. Subsequently, we conducted a literature search in PubMed to finalize the CVDs included in the database. We then manually searched for studies describing relationships between ncRNAs and CVDs in PubMed using appropriate keywords. Finally, we classified the diseases again in the final literature collection; e.g. “Acute heart failure,” “Chronic heart failure” and “Heart failure” were combined in “Heart failure,” and “Acute myocardial infarction,” and “Myocardial infarction” were combined in “Myocardial infarction.” In all of the categories, “Others” was a generalized collective name for unspecified CVDs.

To standardize the data, the design of the database was formalized based on the following screening criteria:

1. Studies in humans and with experimental support, excluding reviews, comments and other types of articles.
2. Studies associated with the disease collected directly.
3. Articles not related to the topic and without appropriate data were excluded.
4. The miRBase (75) was used to unify miRNA names.
5. Various biological annotations were integrated to maximize the information content; for instance, the distribution of miRNA expression across human tissues was covered (76). In particular, we determined the ncRNA as a biomarker if it was concluded or summarized as a candidate/potential biomarker for CVD diagnosis, prognosis and treatment in the article.

### Database implementation

The server for the database was built with MySQL and Apache. PHP was used to connect the database background management system and front-end of the website. Search and browsing results were obtained using HTML and displayed to the user (77).

### CVD–ncRNA network construction and characterization

We mainly explored the network of CVDs and the regulatory effects of ncRNAs from the perspectives of bioinformatics and systems biology to understand regulatory and molecular mechanisms. By analyzing the results collected in the database, we created a CVD–ncRNA binary network and assessed network topologies for different ncRNAs and CVDs. Correlations and distribution patterns of ncRNAs associated with CVDs were examined.

We then performed network function analysis based mainly on miRNAs, which have been investigated most frequently to date (78–80). The collected miRNAs were divided into two categories comprising specific miRNAs related to one disease and universal miRNAs associated with two or more diseases. We aimed to analyze the ability of different miRNAs to regulate diseases by evaluating the number of mRNAs corresponding to miRNAs and the distributions of their functions and signaling pathways. We used MiRNA-BD (81) and four miRNA-related databases, miRTarBase (82), miRecords (83), StarBase (84), and TarBase (85), to collect miRNA target genes. To avoid false positives, we mainly focused on miRNA-target pairs that have been validated by wet-lab experiments, such as q-PCR, from the databases. We employed Cytoscape to present the results. IPA and GO analysis were performed based on all of the miRNA targets before we consulted previous studies regarding enriched pathways to understand the specific processes and effects that might allow them to transmit signals within cells.

As essential genes are considered to be critical for the survival of organisms and indispensable for cellular life (86), we also examined essential genes using the DEG database (87). In methodology, we mapped the targets of miRNAs to records in the DEG database and extracted overlapping genes for further analysis. The ratio of essential genes relative to all target genes was determined to calculate the importance of the miRNAs that we collected.

We summarized related studies and expanded the binary network of CVDs and ncRNAs into ternary networks comprising miRNA–lncRNA–mRNA and miRNA–circRNA–mRNA ternary networks in CVDs, cluster analysis of tissue expression was performed based on the tissue expression profiles from TissueAtlas database.

Finally, statistical analyses were conducted based on biomarkers as indicators of biological processes, pathogenic processes or pharmacological responses to therapeutic interventions (88) using all of the information that we collected. Given the importance of biomarker research, we studied the locations of biomarkers in regulatory networks, analyzed the existence and commonality of ncRNAs that can be used as markers and aimed to determine the heterogeneity of different CVDs at molecular and regulatory levels. Studying biomarkers is important in many scientific fields, and it can facilitate CVD research by providing further directions for research.

#### Geolocation information

Soochow University, 199 Renai Road, Suzhou Industrial Park, Suzhou city, Jiangsu province, China.

## Disclosure statement

The authors report no conflict of interest.

## Data availability statement

The data generated or analyzed in this study are available from the corresponding author upon reasonable request.

## Supplementary Material

Supplementary_1_baz147

Supplementary_2_baz147
