# Supplementary material for: Phenotype–genotype network construction and characterization: a case study of cardiovascular diseases and associated non-coding RNAs
Source: Database (Oxford). 2020 Jan 15;2020:baz147. doi: 10.1093/database/baz147 (PMC6964217; doi:10.1093/database/baz147)
Supplement: Supplementary_1_baz147 [file supplementary_1_baz147.pdf]

Supplementary File 1:

The list of universal miRNAs and specific miRNAs.

	Specific miRNA	Universal miRNA
**1.**	hiv1-miR-H1	hcmv-miR-UL22A-5p
**2.**	HS_202.1	hsa-let-7a-5p
**3.**	hsa-let-7b-3p	hsa-let-7b-5p
**4.**	hsa-let-7d-3p	hsa-let-7c
**5.**	hsa-let-7d-5p	hsa-let-7e-5p
**6.**	hsa-let-7f-1-3p	hsa-let-7f-5p
**7.**	hsa-miR-105-5p	hsa-let-7g-5p
**8.**	hsa-miR-106-5p	hsa-let-7i-5p
**9.**	hsa-miR-106a-5p	hsa-miR-100-5p
**10.**	hsa-miR-10a-3p	hsa-miR-101-3p
**11.**	hsa-miR-10b-3p	hsa-miR-103a-3p
**12.**	hsa-miR-1183	hsa-miR-106b-5p
**13.**	hsa-miR-1202	hsa-miR-107
**14.**	hsa-miR-1207-5p	hsa-miR-10a-5p
**15.**	hsa-miR-122-3p	hsa-miR-10b-5p
**16.**	hsa-miR-1224-5p	hsa-miR-122-5p
**17.**	hsa-miR-1225-3p	hsa-miR-124-3p
**18.**	hsa-miR-1225-5p	hsa-miR-1246
**19.**	hsa-miR-1228-3p	hsa-miR-1254
**20.**	hsa-miR-1228-5p	hsa-miR-125a-5p
**21.**	hsa-miR-1231	hsa-miR-125b-5p
**22.**	hsa-miR-1233-3p	hsa-miR-126-3p
**23.**	hsa-miR-1234-3p	hsa-miR-1274A
**24.**	hsa-miR-1237-3p	hsa-miR-129-5p
**25.**	hsa-miR-1238-3p	hsa-miR-130
**26.**	hsa-miR-124-5p	hsa-miR-1306-3p
**27.**	hsa-miR-1250	hsa-miR-130a-3p
**28.**	hsa-miR-1251	hsa-miR-132-3p
**29.**	hsa-miR-125a-3p	hsa-miR-133
**30.**	hsa-miR-1260a	hsa-miR-133a
**31.**	hsa-miR-126-5p	hsa-miR-133b
**32.**	hsa-miR-1273a	hsa-miR-134
**33.**	hsa-miR-1281	hsa-miR-134-5p
**34.**	hsa-miR-1285-3p	hsa-miR-135a-5p
**35.**	hsa-miR-128b	hsa-miR-135b-5p
**36.**	hsa-miR-1291	hsa-miR-136-5p
**37.**	hsa-miR-1299	hsa-miR-138-5p
**38.**	hsa-miR-130b-3p	hsa-miR-139-5p

**39.**	hsa-miR-133a2	hsa-miR-1-3p
**40.**	hsa-miR-133a-2	hsa-miR-140-3p
**41.**	hsa-miR-135b-3p	hsa-miR-140-5p
**42.**	hsa-miR-137	hsa-miR-142-3p
**43.**	hsa-miR-143-5p	hsa-miR-142-5p
**44.**	hsa-miR-143-MM2	hsa-miR-143-3p
**45.**	hsa-miR-146b-3p	hsa-miR-144-3p
**46.**	hsa-miR-15	hsa-miR-145-5p
**47.**	hsa-miR-150-3p	hsa-miR-146a-5p
**48.**	hsa-miR-151a-3p	hsa-miR-146b-5p
**49.**	hsa-miR-151a-5p	hsa-miR-147a
**50.**	hsa-miR-152	hsa-miR-148a-3p
**51.**	hsa-miR-153	hsa-miR-148b-3p
**52.**	hsa-miR-17-3p	hsa-miR-149-5p
**53.**	hsa-miR-181a-2-3p	hsa-miR-150-5p
**54.**	hsa-miR-181a-3p	hsa-miR-155-5p
**55.**	hsa-miR-181c-3p	hsa-miR-15a-5p
**56.**	hsa-miR-181c-5p	hsa-miR-15b-5p
**57.**	hsa-miR-181d-5p	hsa-miR-16-5p
**58.**	hsa-miR-182-5p	hsa-miR-17-5p
**59.**	hsa-miR-183-3p	hsa-miR-181a-5p
**60.**	hsa-miR-18b-5p	hsa-miR-181b-5p
**61.**	hsa-miR-190a	hsa-miR-184
**62.**	hsa-miR-190b	hsa-miR-185-5p
**63.**	hsa-miR-191-3p	hsa-miR-186-5p
**64.**	hsa-miR-1915-3p	hsa-miR-18a-5p
**65.**	hsa-miR-191-5p	hsa-miR-18b-3p
**66.**	hsa-miR-193-5p	hsa-miR-192-5p
**67.**	hsa-miR-193a-3p	hsa-miR-194-5p
**68.**	hsa-miR-193a-5p	hsa-miR-195-5p
**69.**	hsa-miR-193b-3p	hsa-miR-196a2
**70.**	hsa-miR-193b-5p	hsa-miR-197-3p
**71.**	hsa-miR-195-3p	hsa-miR-199
**72.**	hsa-miR-196a-5p	hsa-miR-199a-3p
**73.**	hsa-miR-196b-5p	hsa-miR-199a-5p
**74.**	hsa-miR-199b-3p	hsa-miR-199b-5p
**75.**	hsa-miR-200a-3p	hsa-miR-19a-3p
**76.**	hsa-miR-200b-5p	hsa-miR-19b-3p
**77.**	hsa-miR-200c-3p	hsa-miR-204-5p
**78.**	hsa-miR-202-3p	hsa-miR-205-5p
**79.**	hsa-miR-208b-3p	hsa-miR-206
**80.**	hsa-miR-20a-3p	hsa-miR-208a
**81.**	hsa-miR-20b-5p	hsa-miR-208a-3p

**82.**	hsa-miR-217	hsa-miR-208b
**83.**	hsa-miR-219a-5p	hsa-miR-20a-5p
**84.**	hsa-miR-220	hsa-miR-210
**85.**	hsa-miR-2222	hsa-miR-211-5p
**86.**	hsa-miR-222-5p	hsa-miR-212-3p
**87.**	hsa-miR-223-5p	hsa-miR-21-3p
**88.**	hsa-miR-22-5p	hsa-miR-214-3p
**89.**	hsa-miR-23	hsa-miR-21-5p
**90.**	hsa-miR-23b-5p	hsa-miR-218-5p
**91.**	hsa-miR-24-2-5p	hsa-miR-221-3p
**92.**	hsa-miR-2861	hsa-miR-222-3p
**93.**	hsa-miR-2909	hsa-miR-223-3p
**94.**	hsa-miR-299-5p	hsa-miR-22-3p
**95.**	hsa-miR-30	hsa-miR-23a-3p
**96.**	hsa-miR-300	hsa-miR-23b-3p
**97.**	hsa-miR-302d-3p	hsa-miR-24-3p
**98.**	hsa-miR-30b-5p	hsa-miR-25-3p
**99.**	hsa-miR-30c-2-3p	hsa-miR-26a-5p
**100.**	hsa-miR-3135b	hsa-miR-26b-5p
**101.**	hsa-miR-3144-5p	hsa-miR-27a-3p
**102.**	hsa-miR-31-5p	hsa-miR-27b-3p
**103.**	hsa-miR-3162-3p	hsa-miR-28-5p
**104.**	hsa-miR-3171	hsa-miR-29
**105.**	hsa-miR-3188	hsa-miR-296-5p
**106.**	hsa-miR-320c	hsa-miR-29a-3p
**107.**	hsa-miR-320e	hsa-miR-29b-3p
**108.**	hsa-miR-322	hsa-miR-29c-3p
**109.**	hsa-miR-32-3p	hsa-miR-30*
**110.**	hsa-miR-324-3p	hsa-miR-301a-3p
**111.**	hsa-miR-32-5p	hsa-miR-302a-3p
**112.**	hsa-miR-338-3p	hsa-miR-302b-3p
**113.**	hsa-miR-339-3p	hsa-miR-302c-3p
**114.**	hsa-miR-33b-3p	hsa-miR-30a-5p
**115.**	hsa-miR-33b-5p	hsa-miR-30c-5p
**116.**	hsa-miR-340-5p	hsa-miR-30d-5p
**117.**	hsa-miR-342-3p	hsa-miR-30e-5p
**118.**	hsa-miR-345-5p	hsa-miR-3130-5p
**119.**	hsa-miR-34b-3p	hsa-miR-3149
**120.**	hsa-miR-34c-5p	hsa-miR-320a
**121.**	hsa-miR-361-5p	hsa-miR-320b
**122.**	hsa-miR-362-3p	hsa-miR-320d
**123.**	hsa-miR-367-3p	hsa-miR-323a-3p
**124.**	hsa-miR-371a-3p	hsa-miR-326

**125.**	hsa-miR-371a-5p	hsa-miR-328
**126.**	hsa-miR-372	hsa-miR-331-3p
**127.**	hsa-miR-373-3p	hsa-miR-335-5p
**128.**	hsa-miR-376a-3p	hsa-miR-339-5p
**129.**	hsa-miR-376c-3p	hsa-miR-33a-5p
**130.**	hsa-miR-377-3p	hsa-miR-340-3p
**131.**	hsa-miR-380-5p	hsa-miR-34a-5p
**132.**	hsa-miR-381-3p	hsa-miR-34b-5p
**133.**	hsa-miR-383	hsa-miR-3615
**134.**	hsa-miR-3908	hsa-miR-363-3p
**135.**	hsa-miR-409-3p	hsa-miR-365a-3p
**136.**	hsa-miR-409-5p	hsa-miR-369-3p
**137.**	hsa-miR-411-5p	hsa-miR-370
**138.**	hsa-miR-421	hsa-miR-375
**139.**	hsa-miR-422	hsa-miR-378a-3p
**140.**	hsa-miR-423-3p	hsa-miR-378a-5p
**141.**	hsa-miR-425-3p	hsa-miR-382-5p
**142.**	hsa-miR-4254	hsa-miR-422a
**143.**	hsa-miR-425-5p	hsa-miR-423-5p
**144.**	hsa-miR-4306	hsa-miR-424-5p
**145.**	hsa-miR-4429	hsa-miR-429
**146.**	hsa-miR-4478	hsa-miR-432-5p
**147.**	hsa-miR-4491	hsa-miR-433
**148.**	hsa-miR-452-5p	hsa-miR-451a
**149.**	hsa-miR-455-3p	hsa-miR-454-3p
**150.**	hsa-miR-455-5p	hsa-miR-483-3p
**151.**	hsa-miR-4643	hsa-miR-483-5p
**152.**	hsa-miR-485-3p	hsa-miR-486-5p
**153.**	hsa-miR-486-3p	hsa-miR-487b
**154.**	hsa-miR-490-3p	hsa-miR-489
**155.**	hsa-miR-491-3p	hsa-miR-493-5p
**156.**	hsa-miR-491-5p	hsa-miR-494
**157.**	hsa-miR-492	hsa-miR-495-3p
**158.**	hsa-miR-497-3p	hsa-miR-497-5p
**159.**	hsa-miR-498	hsa-miR-499a-5p
**160.**	hsa-miR-499a-3p	hsa-miR-500a-3p
**161.**	hsa-miR-503-5p	hsa-miR-519e-5p
**162.**	hsa-miR-507	hsa-miR-523-3p
**163.**	hsa-miR-508-5p	hsa-miR-532-5p
**164.**	hsa-miR-512-5p	hsa-miR-622
**165.**	hsa-miR-515-5p	hsa-miR-625-5p
**166.**	hsa-miR-516a-5p	hsa-miR-636
**167.**	hsa-miR-518f-3p	hsa-miR-720

**168.**	hsa-miR-520b	hsa-miR-770-5p
**169.**	hsa-miR-520c-3p	hsa-miR-877-3p
**170.**	hsa-miR-520d-3p	hsa-miR-92a-3p
**171.**	hsa-miR-520d-5p	hsa-miR-9-5p
**172.**	hsa-miR-520h	hsa-miR-96-5p
**173.**	hsa-miR-524-5p	hsa-miR-98-5p
**174.**	hsa-miR-525-5p	hsa-miR-99b-5p
**175.**	hsa-miR-526b-5p	hsv1-miR-H2-3p
**176.**	hsa-miR-542-3p	
**177.**	hsa-miR-544a	
**178.**	hsa-miR-545-5p	
**179.**	hsa-miR-548c-3p	
**180.**	hsa-miR-550a-3p	
**181.**	hsa-miR-5571-5p	
**182.**	hsa-miR-558	
**183.**	hsa-miR-563	
**184.**	hsa-miR-566	
**185.**	hsa-miR-574-3p	
**186.**	hsa-miR-574-5p	
**187.**	hsa-miR-575	
**188.**	hsa-miR-59	
**189.**	hsa-miR-590-5p	
**190.**	hsa-miR-598	
**191.**	hsa-miR-602	
**192.**	hsa-miR-605	
**193.**	hsa-miR-618	
**194.**	hsa-miR-624-5p	
**195.**	hsa-miR-631	
**196.**	hsa-miR-638	
**197.**	hsa-miR-639	
**198.**	hsa-miR-646	
**199.**	hsa-miR-650	
**200.**	hsa-miR-652-3p	
**201.**	hsa-miR-656	
**202.**	hsa-miR-657	
**203.**	hsa-miR-660-3p	
**204.**	hsa-miR-660-5p	
**205.**	hsa-miR-663a	
**206.**	hsa-miR-663b	
**207.**	hsa-miR-664a-3p	
**208.**	hsa-miR-665	
**209.**	hsa-miR-671-5p	
**210.**	hsa-miR-675-5p	

**211.**	hsa-miR-708-5p	
**212.**	hsa-miR-7-1-3p	
**213.**	hsa-miR-758-3p	
**214.**	hsa-miR-765	
**215.**	hsa-miR-802	
**216.**	hsa-miR-873-5p	
**217.**	hsa-miR-874-3p	
**218.**	hsa-miR-875-3p	
**219.**	hsa-miR-886-3p	
**220.**	hsa-miR-892a	
**221.**	hsa-miR-892b	
**222.**	hsa-miR-92b-3p	
**223.**	hsa-miR-93-3p	
**224.**	hsa-miR-93-5p	
**225.**	hsa-miR-939-5p	
**226.**	hsa-miR-940	
**227.**	hsa-miR-99a-5p	
**228.**	mmu_let_7d	
**229.**	mmu_miR_129_3p	
**230.**	mmu_miR_17_3p	
**231.**	mmu_miR_215	
**232.**	mmu_miR_292_3p	
**233.**	mmu_miR_294	
**234.**	mmu_miR_295	
**235.**	mmu_miR_297	
**236.**	mmu_miR_300	
**237.**	mmu_miR_322	
**238.**	mmu_miR_330	
**239.**	mmu_miR_341	
**240.**	mmu-miR-192	
**241.**	mmu-miR-325	
**242.**	N1	
**243.**	N2	
**244.**	pRe-miR-138	
**245.**	pRe-miR-146a	
**246.**	pre-miR-27a	
**247.**	pRe-miR-499	
**248.**	Rno_miR_297	
**249.**	Rno_miR_333	
**250.**	Rno_miR_343	
**251.**	Rno-miR-352	
