# Supplementary material for: Phenotype–genotype network construction and characterization: a case study of cardiovascular diseases and associated non-coding RNAs
Source: Database (Oxford). 2020 Jan 15;2020:baz147. doi: 10.1093/database/baz147 (PMC6964217; doi:10.1093/database/baz147)
Supplement: Supplementary_2_baz147 [file supplementary_2_baz147.pdf]

Supplementary File 2:

IPA enrichment pathways of the targets of universal miRNAs and specific miRNAs

**Specific miRNAs**						
Ingenuity Canonical Pathways	-log(p)	p	adjusted p (FDR)	adjusted p (bonferroni)	adjusted p (BH)	Ratio
Small Cell Lung Cancer Signaling	3.26	0.00055	0.072013	0.222015	0.072013	0.824
Production of Nitric Oxide and Reactive Oxygen Species in Macrophages	3.26	0.00055	0.072013	0.222015	0.072013	0.824
Pancreatic Adenocarcinoma Signaling	3.11	0.000776	0.072013	0.313604	0.072013	0.789
B Cell Receptor Signaling	3.11	0.000776	0.072013	0.313604	0.072013	0.789
UVB-Induced MAPK Signaling	3.05	0.000891	0.072013	0.360065	0.072013	0.909
Tec Kinase Signaling	2.63	0.002344	0.136195	0.947068	0.136195	0.8
Role of Tissue Factor in Cancer	2.51	0.00309	0.136195	1	0.136195	0.765
UVA-Induced MAPK Signaling	2.47	0.003388	0.136195	1	0.136195	0.833
Sumoylation Pathway	2.47	0.003388	0.136195	1	0.136195	0.833
HGF Signaling	2.43	0.003715	0.136195	1	0.136195	0.737
ILK Signaling	2.36	0.004365	0.136195	1	0.136195	0.714
LPS-stimulated MAPK Signaling	2.33	0.004677	0.136195	1	0.136195	0.786
PI3K/AKT Signaling	2.33	0.004677	0.136195	1	0.136195	0.786
Prostate Cancer Signaling	2.23	0.005888	0.136195	1	0.136195	0.75
RANK Signaling in Osteoclasts	2.23	0.005888	0.136195	1	0.136195	0.75
Molecular Mechanisms of Cancer	2.2	0.00631	0.136195	1	0.136195	0.6
Non-Small Cell Lung Cancer Signaling	2.16	0.006918	0.136195	1	0.136195	0.722
EIF2 Signaling	2.16	0.006918	0.136195	1	0.136195	0.722
Neurotrophin/TRK Signaling	2.16	0.006918	0.136195	1	0.136195	0.722
PEDF Signaling	2.15	0.007079	0.136195	1	0.136195	0.818
Death Receptor Signaling	2.15	0.007079	0.136195	1	0.136195	0.818
IGF-1 Signaling	2.1	0.007943	0.139642	1	0.139642	0.7
Relaxin Signaling	2.03	0.009333	0.139642	1	0.139642	0.769
FLT3 Signaling in Hematopoietic Progenitor Cells	2.03	0.009333	0.139642	1	0.139642	0.769
Myc Mediated Apoptosis Signaling	2.03	0.009333	0.139642	1	0.139642	0.769
SAPK/JNK Signaling	2.03	0.009333	0.139642	1	0.139642	0.769
IL-7 Signaling Pathway	2.03	0.009333	0.139642	1	0.139642	0.769
Estrogen-Dependent Breast Cancer Signaling	1.95	0.01122	0.143836	1	0.143836	0.733
Activation of IRF by Cytosolic Pattern Recognition Receptors	1.93	0.011749	0.143836	1	0.143836	1
TNFR1 Signaling	1.93	0.011749	0.143836	1	0.143836	1
TNFR2 Signaling	1.93	0.011749	0.143836	1	0.143836	1

Inhibition of Angiogenesis by TSP1	1.93	0.011749	0.143836	1	0.143836	1
iNOS Signaling	1.93	0.011749	0.143836	1	0.143836	1
NGF Signaling	1.89	0.012882	0.148701	1	0.148701	0.706
Telomerase Signaling	1.89	0.012882	0.148701	1	0.148701	0.706
Germ Cell-Sertoli Cell Junction Signaling	1.85	0.014125	0.158518	1	0.158518	0.684
RAR Activation	1.83	0.014791	0.161503	1	0.161503	0.667
EGF Signaling	1.74	0.018197	0.173903	1	0.173903	0.75
Apoptosis Signaling	1.74	0.018197	0.173903	1	0.173903	0.75
IL-17 Signaling	1.68	0.020893	0.173903	1	0.173903	0.714
P2Y Purigenic Receptor Signaling Pathway	1.68	0.020893	0.173903	1	0.173903	0.714
T Cell Receptor Signaling	1.68	0.020893	0.173903	1	0.173903	0.714
IL-10 Signaling	1.66	0.021878	0.173903	1	0.173903	0.857
4-1BB Signaling in T Lymphocytes	1.66	0.021878	0.173903	1	0.173903	0.857
Retinoic acid Mediated Apoptosis Signaling	1.66	0.021878	0.173903	1	0.173903	0.857
Toll-like Receptor Signaling	1.66	0.021878	0.173903	1	0.173903	0.857
Apelin Pancreas Signaling Pathway	1.66	0.021878	0.173903	1	0.173903	0.857
14-3-3-mediated Signaling	1.64	0.022909	0.173903	1	0.173903	0.688
IL-12 Signaling and Production in Macrophages	1.64	0.022909	0.173903	1	0.173903	0.688
CXCR4 Signaling	1.64	0.022909	0.173903	1	0.173903	0.688
PDGF Signaling	1.64	0.022909	0.173903	1	0.173903	0.688
AMPK Signaling	1.61	0.024547	0.173903	1	0.173903	0.667
Sertoli Cell-Sertoli Cell Junction Signaling	1.61	0.024547	0.173903	1	0.173903	0.667
p38 MAPK Signaling	1.61	0.024547	0.173903	1	0.173903	0.667
IL-6 Signaling	1.61	0.024547	0.173903	1	0.173903	0.667
Aryl Hydrocarbon Receptor Signaling	1.6	0.025119	0.173903	1	0.173903	0.65
Ovarian Cancer Signaling	1.58	0.026303	0.173903	1	0.173903	0.6
Endocannabinoid Cancer Inhibition Pathway	1.58	0.026303	0.173903	1	0.173903	0.636
Role of PKR in Interferon Induction and Antiviral Response	1.54	0.02884	0.173903	1	0.173903	1
B Cell Activating Factor Signaling	1.54	0.02884	0.173903	1	0.173903	1
April Mediated Signaling	1.54	0.02884	0.173903	1	0.173903	1
Unfolded protein response	1.54	0.02884	0.173903	1	0.173903	1
CD27 Signaling in Lymphocytes	1.54	0.02884	0.173903	1	0.173903	0.778
Induction of Apoptosis by HIV1	1.54	0.02884	0.173903	1	0.173903	0.778
ATM Signaling	1.54	0.02884	0.173903	1	0.173903	0.778
FGF Signaling	1.54	0.02884	0.173903	1	0.173903	0.778
Cancer Drug Resistance By Drug Efflux	1.54	0.02884	0.173903	1	0.173903	0.778

Thrombopoietin Signaling	1.46	0.034674	0.187018	1	0.187018	0.727
GDNF Family Ligand-Receptor Interactions	1.46	0.034674	0.187018	1	0.187018	0.727
UVC-Induced MAPK Signaling	1.46	0.034674	0.187018	1	0.187018	0.727
IL-2 Signaling	1.46	0.034674	0.187018	1	0.187018	0.727
IL-4 Signaling	1.46	0.034674	0.187018	1	0.187018	0.727
CD40 Signaling	1.42	0.038019	0.187018	1	0.187018	0.692
Aldosterone Signaling in Epithelial Cells	1.42	0.038019	0.187018	1	0.187018	0.692
Thyroid Cancer Signaling	1.42	0.038019	0.187018	1	0.187018	0.692
Rac Signaling	1.42	0.038019	0.187018	1	0.187018	0.692
PI3K Signaling in B Lymphocytes	1.42	0.038019	0.187018	1	0.187018	0.692
ErbB2-ErbB3 Signaling	1.42	0.038019	0.187018	1	0.187018	0.692
TGF-β Signaling	1.42	0.038019	0.187018	1	0.187018	0.692
BMP signaling pathway	1.42	0.038019	0.187018	1	0.187018	0.692
p53 Signaling	1.4	0.039811	0.187018	1	0.187018	0.667
Ceramide Signaling	1.4	0.039811	0.187018	1	0.187018	0.667
Type I Diabetes Mellitus Signaling	1.4	0.039811	0.187018	1	0.187018	0.667
Acute Myeloid Leukemia Signaling	1.4	0.039811	0.187018	1	0.187018	0.667
Breast Cancer Regulation by Stathmin1	1.4	0.039811	0.187018	1	0.187018	0.667
ErbB Signaling	1.4	0.039811	0.187018	1	0.187018	0.667
ERK/MAPK Signaling	1.39	0.040738	0.187324	1	0.187324	0.586
HIF1α Signaling	1.38	0.041687	0.187324	1	0.187324	0.647
Glioma Signaling	1.38	0.041687	0.187324	1	0.187324	0.647
GNRH Signaling	1.37	0.042658	0.187324	1	0.187324	0.632
Cardiac Hypertrophy Signaling	1.37	0.042658	0.187324	1	0.187324	0.619
Osteoarthritis Pathway	1.37	0.042658	0.187324	1	0.187324	0.619
IL-1 Signaling	1.33	0.046774	0.198911	1	0.198911	0.833
OX40 Signaling Pathway	1.33	0.046774	0.198911	1	0.198911	0.833
IL-17A Signaling in Gastric Cells	1.33	0.046774	0.198911	1	0.198911	0.833
Colorectal Cancer Metastasis Signaling	1.28	0.052481	0.220856	1	0.220856	0.556
Mitochondrial Dysfunction	1.25	0.056234	0.224937	1	0.224937	0.75
FcγRIIB Signaling in B Lymphocytes	1.25	0.056234	0.224937	1	0.224937	0.75
CCR5 Signaling in Macrophages	1.25	0.056234	0.224937	1	0.224937	0.75
Docosahexaenoic Acid (DHA) Signaling	1.25	0.056234	0.224937	1	0.224937	0.75
Hypoxia Signaling in the Cardiovascular System	1.25	0.056234	0.224937	1	0.224937	0.75
IL-8 Signaling	1.22	0.060256	0.234874	1	0.234874	0.571
Amyotrophic Lateral Sclerosis Signaling	1.2	0.063096	0.234874	1	0.234874	0.7
CNTF Signaling	1.2	0.063096	0.234874	1	0.234874	0.7
Cdc42 Signaling	1.2	0.063096	0.234874	1	0.234874	0.7
Glioma Invasiveness Signaling	1.2	0.063096	0.234874	1	0.234874	0.7
ErbB4 Signaling	1.2	0.063096	0.234874	1	0.234874	0.7
GM-CSF Signaling	1.2	0.063096	0.234874	1	0.234874	0.7

PTEN Signaling	1.18	0.066069	0.234874	1	0.234874	0.591
mTOR Signaling	1.18	0.066069	0.234874	1	0.234874	0.667
HER-2 Signaling in Breast Cancer	1.18	0.066069	0.234874	1	0.234874	0.667
IL-3 Signaling	1.17	0.067608	0.234874	1	0.234874	0.643
Corticotropin Releasing Hormone Signaling	1.17	0.067608	0.234874	1	0.234874	0.643
Chronic Myeloid Leukemia Signaling	1.17	0.067608	0.234874	1	0.234874	0.643
PPAR Signaling	1.16	0.069183	0.234874	1	0.234874	0.611
Leukocyte Extravasation Signaling	1.16	0.069183	0.234874	1	0.234874	0.625
Erythropoietin Signaling	1.16	0.069183	0.234874	1	0.234874	0.625
Renal Cell Carcinoma Signaling	1.16	0.069183	0.234874	1	0.234874	0.625
PKCθ Signaling in T Lymphocytes	1.16	0.069183	0.234874	1	0.234874	0.625
Regulation of the Epithelial-Mesenchymal Transition Pathway	1.06	0.087096	0.293224	1	0.293224	0.552
Glioblastoma Multiforme Signaling	1.03	0.093325	0.311257	1	0.311257	0.56
MSP-RON Signaling Pathway	1.02	0.095499	0.311257	1	0.311257	0.8
Sirtuin Signaling Pathway	0.996	0.100925	0.311257	1	0.311257	0.571
Mouse Embryonic Stem Cell Pluripotency	0.983	0.103992	0.311257	1	0.311257	0.579
Hepatic Cholestasis	0.967	0.107895	0.311257	1	0.311257	0.588
Clathrin-mediated Endocytosis Signaling	0.967	0.107895	0.311257	1	0.311257	0.588
Lymphotoxin β Receptor Signaling	0.967	0.107895	0.311257	1	0.311257	0.714
Cholecystokinin/Gastrin-mediated Signaling	0.967	0.107895	0.311257	1	0.311257	0.588
Prolactin Signaling	0.967	0.107895	0.311257	1	0.311257	0.588
Bladder Cancer Signaling	0.967	0.107895	0.311257	1	0.311257	0.588
CD28 Signaling in T Helper Cells	0.955	0.110917	0.311257	1	0.311257	0.6
Melanocyte Development and Pigmentation Signaling	0.955	0.110917	0.311257	1	0.311257	0.6
Endometrial Cancer Signaling	0.955	0.110917	0.311257	1	0.311257	0.6
Type II Diabetes Mellitus Signaling	0.955	0.110917	0.311257	1	0.311257	0.6
Tight Junction Signaling	0.951	0.111944	0.311257	1	0.311257	0.667
Regulation of eIF4 and p70S6K Signaling	0.951	0.111944	0.311257	1	0.311257	0.667
PCP pathway	0.951	0.111944	0.311257	1	0.311257	0.667
IL-15 Signaling	0.947	0.11298	0.311257	1	0.311257	0.615
Dendritic Cell Maturation	0.947	0.11298	0.311257	1	0.311257	0.615
FAK Signaling	0.947	0.11298	0.311257	1	0.311257	0.615
PAK Signaling	0.947	0.11298	0.311257	1	0.311257	0.615
Role of IL-17A in Arthritis	0.947	0.11298	0.311257	1	0.311257	0.615
Apelin Endothelial Signaling Pathway	0.947	0.11298	0.311257	1	0.311257	0.615
CREB Signaling in Neurons	0.943	0.114025	0.311257	1	0.311257	0.636

Melanoma Signaling	0.943	0.114025	0.311257	1	0.311257	0.636
IL-17A Signaling in Airway Cells	0.943	0.114025	0.311257	1	0.311257	0.636
Chemokine Signaling	0.943	0.114025	0.311257	1	0.311257	0.636
SPINK1 General Cancer Pathway	0.943	0.114025	0.311257	1	0.311257	0.636
Axonal Guidance Signaling	0.924	0.119124	0.322994	1	0.322994	0.512
Role of Osteoblasts, Osteoclasts and Chondrocytes in Rheumatoid Arthritis	0.91	0.123027	0.331352	1	0.331352	0.536
Neuroinflammation Signaling Pathway	0.903	0.125026	0.334506	1	0.334506	0.513
Adrenomedullin signaling pathway	0.866	0.136144	0.361858	1	0.361858	0.542
Glucocorticoid Receptor Signaling	0.857	0.138995	0.36702	1	0.36702	0.514
NRF2-mediated Oxidative Stress Response	0.845	0.142889	0.36769	1	0.36769	0.545
Wnt/β-catenin Signaling	0.845	0.142889	0.36769	1	0.36769	0.545
NF-κB Signaling	0.845	0.142889	0.36769	1	0.36769	0.545
Ephrin Receptor Signaling	0.845	0.142889	0.36769	1	0.36769	0.545
HMGB1 Signaling	0.785	0.164059	0.399683	1	0.399683	0.562
Human Embryonic Stem Cell Pluripotency	0.785	0.164059	0.399683	1	0.399683	0.562
Systemic Lupus Erythematosus Signaling	0.785	0.164059	0.399683	1	0.399683	0.562
JAK/Stat Signaling	0.785	0.164059	0.399683	1	0.399683	0.562
Circadian Rhythm Signaling	0.77	0.169824	0.399683	1	0.399683	1
Cytotoxic T Lymphocyte-mediated Apoptosis of Target Cells	0.77	0.169824	0.399683	1	0.399683	1
Polyamine Regulation in Colon Cancer	0.77	0.169824	0.399683	1	0.399683	1
Extrinsic Prothrombin Activation Pathway	0.77	0.169824	0.399683	1	0.399683	1
Tumoricidal Function of Hepatic Natural Killer Cells	0.77	0.169824	0.399683	1	0.399683	1
Estrogen-mediated S-phase Entry	0.77	0.169824	0.399683	1	0.399683	1
HIPPO signaling	0.77	0.169824	0.399683	1	0.399683	1
Interferon Signaling	0.77	0.169824	0.399683	1	0.399683	1
Endoplasmic Reticulum Stress Pathway	0.77	0.169824	0.399683	1	0.399683	1
SPINK1 Pancreatic Cancer Pathway	0.77	0.169824	0.399683	1	0.399683	1
MIF Regulation of Innate Immunity	0.764	0.172187	0.399683	1	0.399683	0.571
Growth Hormone Signaling	0.764	0.172187	0.399683	1	0.399683	0.571
VEGF Signaling	0.764	0.172187	0.399683	1	0.399683	0.571
Regulation of IL-2 Expression in Activated and Anergic T Lymphocytes	0.747	0.179061	0.399683	1	0.399683	0.583
G-Protein Coupled Receptor Signaling	0.74	0.18197	0.399683	1	0.399683	0.52
Leptin Signaling in Obesity	0.73	0.186209	0.399683	1	0.399683	0.6
eNOS Signaling	0.73	0.186209	0.399683	1	0.399683	0.6
Opioid Signaling Pathway	0.717	0.191867	0.399683	1	0.399683	0.522

NF-κB Activation by Viruses	0.714	0.193197	0.399683	1	0.399683	0.625
IL-9 Signaling	0.714	0.193197	0.399683	1	0.399683	0.625
Virus Entry via Endocytic Pathways	0.714	0.193197	0.399683	1	0.399683	0.625
Neuropathic Pain Signaling In Dorsal Horn Neurons	0.714	0.193197	0.399683	1	0.399683	0.625
G Beta Gamma Signaling	0.714	0.193197	0.399683	1	0.399683	0.625
Paxillin Signaling	0.714	0.193197	0.399683	1	0.399683	0.625
Ephrin B Signaling	0.714	0.193197	0.399683	1	0.399683	0.625
Regulation of Cellular Mechanics by Calpain Protease	0.714	0.193197	0.399683	1	0.399683	0.625
Apelin Liver Signaling Pathway	0.714	0.193197	0.399683	1	0.399683	0.625
IL-17A Signaling in Fibroblasts	0.712	0.194089	0.399683	1	0.399683	0.75
Differential Regulation of Cytokine Production in Macrophages and T Helper Cells by IL-17A and IL-17F	0.712	0.194089	0.399683	1	0.399683	0.75
Differential Regulation of Cytokine Production in Intestinal Epithelial Cells by IL-17A and IL-17F	0.712	0.194089	0.399683	1	0.399683	0.75
GADD45 Signaling	0.712	0.194089	0.399683	1	0.399683	0.75
Cardiac β-adrenergic Signaling	0.712	0.194089	0.399683	1	0.399683	0.75
Agrin Interactions at Neuromuscular Junction	0.708	0.195884	0.399683	1	0.399683	0.667
Androgen Signaling	0.708	0.195884	0.399683	1	0.399683	0.667
Sphingosine-1-phosphate Signaling	0.708	0.195884	0.399683	1	0.399683	0.667
Role of p14/p19ARF in Tumor Suppression	0.708	0.195884	0.399683	1	0.399683	0.667
Cell Cycle: G1/S Checkpoint Regulation	0.708	0.195884	0.399683	1	0.399683	0.667
Renin-Angiotensin Signaling	0.693	0.202768	0.41165	1	0.41165	0.524
Role of NANOG in Mammalian Embryonic Stem Cell Pluripotency	0.668	0.214783	0.429566	1	0.429566	0.526
Role of MAPK Signaling in the Pathogenesis of Influenza	0.668	0.214783	0.429566	1	0.429566	0.526
Insulin Receptor Signaling	0.668	0.214783	0.429566	1	0.429566	0.526
PPARα/RXRα Activation	0.642	0.228034	0.449394	1	0.449394	0.529
Gα12/13 Signaling	0.642	0.228034	0.449394	1	0.449394	0.529
Integrin Signaling	0.642	0.228034	0.449394	1	0.449394	0.529
Role of Macrophages, Fibroblasts and Endothelial Cells in Rheumatoid Arthritis	0.635	0.231739	0.454479	1	0.454479	0.486
Adipogenesis pathway	0.616	0.242103	0.47251	1	0.47251	0.533
Huntington's Disease Signaling	0.606	0.247742	0.481192	1	0.481192	0.5
Neuregulin Signaling	0.59	0.25704	0.492152	1	0.492152	0.538
TR/RXR Activation	0.59	0.25704	0.492152	1	0.492152	0.538

Reelin Signaling in Neurons	0.59	0.25704	0.492152	1	0.492152	0.538
Endocannabinoid Developing Neuron Pathway	0.58	0.263027	0.50124	1	0.50124	0.5
PXR/RXR Activation	0.562	0.274157	0.513958	1	0.513958	0.545
Basal Cell Carcinoma Signaling	0.562	0.274157	0.513958	1	0.513958	0.545
Role of JAK1 and JAK3 in γc Cytokine Signaling	0.562	0.274157	0.513958	1	0.513958	0.545
Endothelin-1 Signaling	0.561	0.274789	0.513958	1	0.513958	0.483
Role of Pattern Recognition Receptors in Recognition of Bacteria and Viruses	0.533	0.293089	0.538219	1	0.538219	0.556
Angiopoietin Signaling	0.533	0.293089	0.538219	1	0.538219	0.556
iCOS-iCOSL Signaling in T Helper Cells	0.533	0.293089	0.538219	1	0.538219	0.556
Role of PI3K/AKT Signaling in the Pathogenesis of Influenza	0.533	0.293089	0.538219	1	0.538219	0.556
Role of NFAT in Regulation of the Immune Response	0.524	0.299226	0.544538	1	0.544538	0.5
Signaling by Rho Family GTPases	0.524	0.299226	0.544538	1	0.544538	0.5
Synaptic Long Term Potentiation	0.503	0.314051	0.558928	1	0.558928	0.571
Oncostatin M Signaling	0.503	0.314051	0.558928	1	0.558928	0.571
Melatonin Signaling	0.503	0.314051	0.558928	1	0.558928	0.571
Role of IL-17F in Allergic Inflammatory Airway Diseases	0.503	0.314051	0.558928	1	0.558928	0.571
GP6 Signaling Pathway	0.503	0.314051	0.558928	1	0.558928	0.571
Thrombin Signaling	0.496	0.319154	0.563049	1	0.563049	0.5
p70S6K Signaling	0.496	0.319154	0.563049	1	0.563049	0.5
Hepatic Fibrosis / Hepatic Stellate Cell Activation	0.478	0.33266	0.577409	1	0.577409	0.467
TREM1 Signaling	0.47	0.338844	0.577409	1	0.577409	0.6
Graft-versus-Host Disease Signaling	0.47	0.338844	0.577409	1	0.577409	0.6
Role of Hypercytokinemia/hyperchemokinemia in the Pathogenesis of Influenza	0.47	0.338844	0.577409	1	0.577409	0.6
Cyclins and Cell Cycle Regulation	0.47	0.338844	0.577409	1	0.577409	0.6
Ephrin A Signaling	0.47	0.338844	0.577409	1	0.577409	0.6
Protein Ubiquitination Pathway	0.47	0.338844	0.577409	1	0.577409	0.6
Amyloid Processing	0.47	0.338844	0.577409	1	0.577409	0.6
Th2 Pathway	0.466	0.341979	0.577409	1	0.577409	0.5
Fc Epsilon RI Signaling	0.456	0.349945	0.577409	1	0.577409	0.476
VEGF Family Ligand-Receptor Interactions	0.456	0.349945	0.577409	1	0.577409	0.476
fMLP Signaling in Neutrophils	0.434	0.368129	0.577409	1	0.577409	0.5
Hereditary Breast Cancer Signaling	0.434	0.368129	0.577409	1	0.577409	0.5
Antioxidant Action of Vitamin C	0.434	0.368129	0.577409	1	0.577409	0.5

Gαq Signaling	0.434	0.368129	0.577409	1	0.577409	0.5
Nitric Oxide Signaling in the Cardiovascular System	0.434	0.368129	0.577409	1	0.577409	0.5
IL-22 Signaling	0.432	0.369828	0.577409	1	0.577409	0.667
Semaphorin Signaling in Neurons	0.432	0.369828	0.577409	1	0.577409	0.667
Mitotic Roles of Polo-Like Kinase	0.432	0.369828	0.577409	1	0.577409	0.667
Pathogenesis of Multiple Sclerosis	0.432	0.369828	0.577409	1	0.577409	0.667
Actin Nucleation by ARP-WASP Complex	0.432	0.369828	0.577409	1	0.577409	0.667
nNOS Signaling in Skeletal Muscle Cells	0.432	0.369828	0.577409	1	0.577409	0.667
autophagy	0.432	0.369828	0.577409	1	0.577409	0.667
Acute Phase Response Signaling	0.427	0.374111	0.577409	1	0.577409	0.462
cAMP-mediated signaling	0.427	0.374111	0.577409	1	0.577409	0.474
Natural Killer Cell Signaling	0.399	0.399025	0.577409	1	0.577409	0.5
CDK5 Signaling	0.399	0.399025	0.577409	1	0.577409	0.5
Role of NFAT in Cardiac Hypertrophy	0.398	0.399945	0.577409	1	0.577409	0.471
Role of RIG1-like Receptors in Antiviral Innate Immunity	0.384	0.413048	0.577409	1	0.577409	1
Mechanisms of Viral Exit from Host Cells	0.384	0.413048	0.577409	1	0.577409	1
Cell Cycle Regulation by BTG Family Proteins	0.384	0.413048	0.577409	1	0.577409	1
TWEAK Signaling	0.384	0.413048	0.577409	1	0.577409	1
Role of JAK1, JAK2 and TYK2 in Interferon Signaling	0.384	0.413048	0.577409	1	0.577409	1
NAD Biosynthesis from 2-amino-3-carboxymuconate Semialdehyde	0.384	0.413048	0.577409	1	0.577409	1
NAD biosynthesis II (from tryptophan)	0.384	0.413048	0.577409	1	0.577409	1
Glutathione Redox Reactions I	0.384	0.413048	0.577409	1	0.577409	1
Heme Degradation	0.384	0.413048	0.577409	1	0.577409	1
Tetrahydrofolate Salvage from 5,10-methenyltetrahydrofolate	0.384	0.413048	0.577409	1	0.577409	1
γ-linolenate Biosynthesis II (Animals)	0.384	0.413048	0.577409	1	0.577409	1
Guanine and Guanosine Salvage I	0.384	0.413048	0.577409	1	0.577409	1
Tetrapyrrole Biosynthesis II	0.384	0.413048	0.577409	1	0.577409	1
Oleate Biosynthesis II (Animals)	0.384	0.413048	0.577409	1	0.577409	1
5-aminoimidazole Ribonucleotide Biosynthesis I	0.384	0.413048	0.577409	1	0.577409	1
S-methyl-5'-thioadenosine Degradation II	0.384	0.413048	0.577409	1	0.577409	1
Glycogen Degradation III	0.384	0.413048	0.577409	1	0.577409	1
Calcium Transport I	0.384	0.413048	0.577409	1	0.577409	1
Glycogen Degradation II	0.384	0.413048	0.577409	1	0.577409	1

Mevalonate Pathway I	0.384	0.413048	0.577409	1	0.577409	1
Glutathione Redox Reactions II	0.384	0.413048	0.577409	1	0.577409	1
Heme Biosynthesis II	0.384	0.413048	0.577409	1	0.577409	1
Purine Nucleotides De Novo Biosynthesis II	0.384	0.413048	0.577409	1	0.577409	1
Citrulline-Nitric Oxide Cycle	0.384	0.413048	0.577409	1	0.577409	1
Methionine Degradation I (to Homocysteine)	0.384	0.413048	0.577409	1	0.577409	1
Superpathway of Methionine Degradation	0.384	0.413048	0.577409	1	0.577409	1
S-adenosyl-L-methionine Biosynthesis	0.384	0.413048	0.577409	1	0.577409	1
Cysteine Biosynthesis III (mammalia)	0.384	0.413048	0.577409	1	0.577409	1
Vitamin-C Transport	0.384	0.413048	0.577409	1	0.577409	1
Oxidative Phosphorylation	0.384	0.413048	0.577409	1	0.577409	1
BER pathway	0.384	0.413048	0.577409	1	0.577409	1
Antigen Presentation Pathway	0.384	0.413048	0.577409	1	0.577409	1
Actin Cytoskeleton Signaling	0.367	0.429536	0.592262	1	0.592262	0.467
Agranulocyte Adhesion and Diapedesis	0.367	0.429536	0.592262	1	0.592262	0.467
Granulocyte Adhesion and Diapedesis	0.367	0.429536	0.592262	1	0.592262	0.467
Th1 and Th2 Activation Pathway	0.367	0.429536	0.592262	1	0.592262	0.467
Crosstalk between Dendritic Cells and Natural Killer Cells	0.361	0.435512	0.594415	1	0.594415	0.5
Role of Wnt/GSK-3β Signaling in the Pathogenesis of Influenza	0.361	0.435512	0.594415	1	0.594415	0.5
Estrogen Receptor Signaling	0.361	0.435512	0.594415	1	0.594415	0.5
STAT3 Pathway	0.357	0.439542	0.595889	1	0.595889	0.444
Xenobiotic Metabolism Signaling	0.357	0.439542	0.595889	1	0.595889	0.444
Antiproliferative Role of Somatostatin Receptor 2	0.333	0.464515	0.625547	1	0.625547	0.462
ERK5 Signaling	0.333	0.464515	0.625547	1	0.625547	0.462
LPS/IL-1 Mediated Inhibition of RXR Function	0.317	0.481948	0.629535	1	0.629535	0.444
α-Adrenergic Signaling	0.316	0.483059	0.629535	1	0.629535	0.5
Allograft Rejection Signaling	0.316	0.483059	0.629535	1	0.629535	0.5
Dopamine-DARPP32 Feedback in cAMP Signaling	0.316	0.483059	0.629535	1	0.629535	0.5
Remodeling of Epithelial Adherens Junctions	0.316	0.483059	0.629535	1	0.629535	0.5
Wnt/Ca+ pathway	0.316	0.483059	0.629535	1	0.629535	0.5
GPCR-Mediated Integration of Enteroendocrine Signaling Exemplified by an L Cell	0.316	0.483059	0.629535	1	0.629535	0.5
Phagosome Formation	0.316	0.483059	0.629535	1	0.629535	0.5

Phagosome Maturation	0.316	0.483059	0.629535	1	0.629535	0.5
Apelin Cardiomyocyte Signaling Pathway	0.316	0.483059	0.629535	1	0.629535	0.5
MIF-mediated Glucocorticoid Regulation	0.298	0.503501	0.654065	1	0.654065	0.455
Atherosclerosis Signaling	0.287	0.516416	0.666557	1	0.666557	0.438
Gap Junction Signaling	0.287	0.516416	0.666557	1	0.666557	0.438
Airway Pathology in Chronic Obstructive Pulmonary Disease	0.262	0.547016	0.691634	1	0.691634	0.5
Cardiomyocyte Differentiation via BMP Receptors	0.262	0.547016	0.691634	1	0.691634	0.5
Fatty Acid α-oxidation	0.262	0.547016	0.691634	1	0.691634	0.5
Inhibition of Matrix Metalloproteases	0.262	0.547016	0.691634	1	0.691634	0.5
Cell Cycle: G2/M DNA Damage Checkpoint Regulation	0.262	0.547016	0.691634	1	0.691634	0.5
Sonic Hedgehog Signaling	0.262	0.547016	0.691634	1	0.691634	0.5
Communication between Innate and Adaptive Immune Cells	0.26	0.549541	0.691634	1	0.691634	0.444
Macropinocytosis Signaling	0.26	0.549541	0.691634	1	0.691634	0.444
Superpathway of Inositol Phosphate Compounds	0.256	0.554626	0.693711	1	0.693711	0.429
3-phosphoinositide Biosynthesis	0.256	0.554626	0.693711	1	0.693711	0.429
CCR3 Signaling in Eosinophils	0.252	0.559758	0.697969	1	0.697969	0.421
Th1 Pathway	0.222	0.599791	0.745586	1	0.745586	0.417
Coagulation System	0.215	0.609537	0.748489	1	0.748489	0.429
CTLA4 Signaling in Cytotoxic T Lymphocytes	0.215	0.609537	0.748489	1	0.748489	0.429
Role of Oct4 in Mammalian Embryonic Stem Cell Pluripotency	0.215	0.609537	0.748489	1	0.748489	0.429
Autoimmune Thyroid Disease Signaling	0.215	0.609537	0.748489	1	0.748489	0.429
Regulation of Actin-based Motility by Rho	0.183	0.656145	0.772836	1	0.772836	0.5
Fcγ Receptor-mediated Phagocytosis in Macrophages and Monocytes	0.183	0.656145	0.772836	1	0.772836	0.5
IL-15 Production	0.183	0.656145	0.772836	1	0.772836	0.5
Airway Inflammation in Asthma	0.183	0.656145	0.772836	1	0.772836	0.5
DNA Double-Strand Break Repair by Homologous Recombination	0.183	0.656145	0.772836	1	0.772836	0.5
nNOS Signaling in Neurons	0.183	0.656145	0.772836	1	0.772836	0.5
Prostanoid Biosynthesis	0.183	0.656145	0.772836	1	0.772836	0.5
Superpathway of Citrulline Metabolism	0.183	0.656145	0.772836	1	0.772836	0.5
Triacylglycerol Biosynthesis	0.183	0.656145	0.772836	1	0.772836	0.5
Superpathway of	0.183	0.656145	0.772836	1	0.772836	0.5

Geranylgeranyldiphosphate Biosynthesis I (via Mevalonate)						
Superpathway of Cholesterol Biosynthesis	0.183	0.656145	0.772836	1	0.772836	0.5
Phototransduction Pathway	0.183	0.656145	0.772836	1	0.772836	0.5
Notch Signaling	0.183	0.656145	0.772836	1	0.772836	0.5
Gustation Pathway	0.183	0.656145	0.772836	1	0.772836	0.5
Synaptic Long Term Depression	0	1	1	1	1	0.353
LXR/RXR Activation	0	1	1	1	1	0.389
VDR/RXR Activation	0	1	1	1	1	0.333
FXR/RXR Activation	0	1	1	1	1	0.375
Role of BRCA1 in DNA Damage Response	0	1	1	1	1	0.333
Caveolar-mediated Endocytosis Signaling	0	1	1	1	1	0.4
T Helper Cell Differentiation	0	1	1	1	1	0.286
Role of Cytokines in Mediating Communication between Immune Cells	0	1	1	1	1	0.333
Cellular Effects of Sildenafil (Viagra)	0	1	1	1	1	0.2
Factors Promoting Cardiogenesis in Vertebrates	0	1	1	1	1	0.375
Role of CHK Proteins in Cell Cycle Checkpoint Control	0	1	1	1	1	0.25
G Protein Signaling Mediated by Tubby	0	1	1	1	1	0.333
RhoA Signaling	0	1	1	1	1	0.375
Phospholipase C Signaling	0	1	1	1	1	0.333
Altered T Cell and B Cell Signaling in Rheumatoid Arthritis	0	1	1	1	1	0.333
Protein Kinase A Signaling	0	1	1	1	1	0.409
Neuroprotective Role of THOP1 in Alzheimer's Disease	0	1	1	1	1	0.364
Nur77 Signaling in T Lymphocytes	0	1	1	1	1	0.143
Cell Cycle Control of Chromosomal Replication	0	1	1	1	1	0.25
Role of JAK2 in Hormone-like Cytokine Signaling	0	1	1	1	1	0.125
Role of JAK family kinases in IL-6-type Cytokine Signaling	0	1	1	1	1	0.4
RhoGDI Signaling	0	1	1	1	1	0.375
Netrin Signaling	0	1	1	1	1	0.25
Bupropion Degradation	0	1	1	1	1	0.2
Nicotine Degradation III	0	1	1	1	1	0.167
Dopamine Degradation	0	1	1	1	1	0.333

D-myo-inositol-5-phosphate Metabolism	0	1	1	1	1	0.4
Melatonin Degradation I	0	1	1	1	1	0.167
Pyridoxal 5'-phosphate Salvage Pathway	0	1	1	1	1	0.4
Phospholipases	0	1	1	1	1	0.25
Estrogen Biosynthesis	0	1	1	1	1	0.2
Tryptophan Degradation X (Mammalian, via Tryptamine)	0	1	1	1	1	0.25
D-myo-inositol (1,4,5,6)-Tetrakisphosphate Biosynthesis	0	1	1	1	1	0.4
Nicotine Degradation II	0	1	1	1	1	0.167
Serotonin Degradation	0	1	1	1	1	0.25
D-myo-inositol (3,4,5,6)-tetrakisphosphate Biosynthesis	0	1	1	1	1	0.4
3-phosphoinositide Degradation	0	1	1	1	1	0.4
Adenine and Adenosine Salvage III	0	1	1	1	1	0.333
Salvage Pathways of Pyrimidine Ribonucleotides	0	1	1	1	1	0.364
Histamine Degradation	0	1	1	1	1	0.333
Superpathway of Melatonin Degradation	0	1	1	1	1	0.167
Noradrenaline and Adrenaline Degradation	0	1	1	1	1	0.333
Acetone Degradation I (to Methylglyoxal)	0	1	1	1	1	0.2
Putrescine Degradation III	0	1	1	1	1	0.333
Oxidative Ethanol Degradation III	0	1	1	1	1	0.333
Ethanol Degradation IV	0	1	1	1	1	0.333
Ethanol Degradation II	0	1	1	1	1	0.333
DNA damage-induced 14-3-3 σ Signaling	0	1	1	1	1	0.333
Epithelial Adherens Junction Signaling	0	1	1	1	1	0.385
Gαi Signaling	0	1	1	1	1	0.364
Gαs Signaling	0	1	1	1	1	0.357
Sperm Motility	0	1	1	1	1	0.273
Dopamine Receptor Signaling	0	1	1	1	1	0.333
Glutamate Receptor Signaling	0	1	1	1	1	0.333
GPCR-Mediated Nutrient Sensing in Enteroendocrine Cells	0	1	1	1	1	0.25
Iron homeostasis signaling pathway	0	1	1	1	1	0.364
Th17 Activation Pathway	0	1	1	1	1	0.333
Apelin Adipocyte Signaling Pathway	0	1	1	1	1	0.2

Calcium Signaling	0	1	1	1	1	0.333
Complement System	0	1	1	1	1	0.2
Eicosanoid Signaling	0	1	1	1	1	0.3
**Universal miRNAs**						
Ingenuity Canonical Pathways	-log(p-value)	p-value	adjusted p-value(FDR)	adjusted p-value(bonferroni)	adjusted p-value(BH)	Ratio
Molecular Mechanisms of Cancer	4.18	6.61E-05	0.026317	0.028476	0.026317	0.867
Role of Tissue Factor in Cancer	3.79	0.000162	0.026317	0.0699	0.026317	1
RAR Activation	3.51	0.000309	0.026317	0.133192	0.026317	0.952
ILK Signaling	3.51	0.000309	0.026317	0.133192	0.026317	0.952
p53 Signaling	3.33	0.000468	0.026317	0.201594	0.026317	1
Estrogen-Dependent Breast Cancer Signaling	3.33	0.000468	0.026317	0.201594	0.026317	1
Aryl Hydrocarbon Receptor Signaling	3.3	0.000501	0.026317	0.216012	0.026317	0.95
IGF-1 Signaling	3.3	0.000501	0.026317	0.216012	0.026317	0.95
Colorectal Cancer Metastasis Signaling	3.26	0.00055	0.026317	0.236852	0.026317	0.861
Germ Cell-Sertoli Cell Junction Signaling	3.09	0.000813	0.029194	0.35033	0.029194	0.947
B Cell Receptor Signaling	3.09	0.000813	0.029194	0.35033	0.029194	0.947
Insulin Receptor Signaling	3.09	0.000813	0.029194	0.35033	0.029194	0.947
EIF2 Signaling	2.89	0.001288	0.037184	0.555236	0.037184	0.944
AMPK Signaling	2.89	0.001288	0.037184	0.555236	0.037184	0.944
Myc Mediated Apoptosis Signaling	2.88	0.001318	0.037184	0.568169	0.037184	1
Ovarian Cancer Signaling	2.86	0.00138	0.037184	0.594946	0.037184	0.867
Small Cell Lung Cancer Signaling	2.68	0.002089	0.043859	0.900487	0.043859	0.941
Gα12/13 Signaling	2.68	0.002089	0.043859	0.900487	0.043859	0.941
mTOR Signaling	2.66	0.002188	0.043859	0.942925	0.043859	1
UVA-Induced MAPK Signaling	2.66	0.002188	0.043859	0.942925	0.043859	1
Sumoylation Pathway	2.66	0.002188	0.043859	0.942925	0.043859	1
Glioblastoma Multiforme Signaling	2.65	0.002239	0.043859	0.964889	0.043859	0.88
Role of Macrophages, Fibroblasts and Endothelial Cells in Rheumatoid Arthritis	2.51	0.00309	0.054891	1	0.054891	0.829
14-3-3-mediated Signaling	2.48	0.003311	0.054891	1	0.054891	0.938
Prostate Cancer Signaling	2.48	0.003311	0.054891	1	0.054891	0.938
PDGF Signaling	2.48	0.003311	0.054891	1	0.054891	0.938
PEDF Signaling	2.43	0.003715	0.05719	1	0.05719	1
UVB-Induced MAPK Signaling	2.43	0.003715	0.05719	1	0.05719	1
Ceramide Signaling	2.28	0.005248	0.074016	1	0.074016	0.933
Endometrial Cancer Signaling	2.28	0.005248	0.074016	1	0.074016	0.933
HGF Signaling	2.26	0.005495	0.074016	1	0.074016	0.895

Pancreatic Adenocarcinoma Signaling	2.26	0.005495	0.074016	1	0.074016	0.895
Ephrin Receptor Signaling	2.12	0.007586	0.085355	1	0.085355	0.864
Regulation of the Epithelial-Mesenchymal Transition Pathway	2.11	0.007762	0.085355	1	0.085355	0.828
LPS-stimulated MAPK Signaling	2.08	0.008318	0.085355	1	0.085355	0.929
IL-17 Signaling	2.08	0.008318	0.085355	1	0.085355	0.929
Chronic Myeloid Leukemia Signaling	2.08	0.008318	0.085355	1	0.085355	0.929
PI3K/AKT Signaling	2.08	0.008318	0.085355	1	0.085355	0.929
Non-Small Cell Lung Cancer Signaling	2.08	0.008318	0.085355	1	0.085355	0.889
Sertoli Cell-Sertoli Cell Junction Signaling	2.08	0.008318	0.085355	1	0.085355	0.889
PPAR Signaling	2.08	0.008318	0.085355	1	0.085355	0.889
IL-6 Signaling	2.08	0.008318	0.085355	1	0.085355	0.889
ATM Signaling	1.98	0.010471	0.094108	1	0.094108	1
Cardiac Hypertrophy Signaling	1.95	0.01122	0.094108	1	0.094108	0.857
Osteoarthritis Pathway	1.95	0.01122	0.094108	1	0.094108	0.857
Sirtuin Signaling Pathway	1.95	0.01122	0.094108	1	0.094108	0.857
PPARα/RXRα Activation	1.9	0.012589	0.094108	1	0.094108	0.882
HIF1α Signaling	1.9	0.012589	0.094108	1	0.094108	0.882
Prolactin Signaling	1.9	0.012589	0.094108	1	0.094108	0.882
Glioma Signaling	1.9	0.012589	0.094108	1	0.094108	0.882
Production of Nitric Oxide and Reactive Oxygen Species in Macrophages	1.9	0.012589	0.094108	1	0.094108	0.882
NGF Signaling	1.9	0.012589	0.094108	1	0.094108	0.882
Telomerase Signaling	1.9	0.012589	0.094108	1	0.094108	0.882
TR/RXR Activation	1.89	0.012882	0.094108	1	0.094108	0.923
Thyroid Cancer Signaling	1.89	0.012882	0.094108	1	0.094108	0.923
ErbB2-ErbB3 Signaling	1.89	0.012882	0.094108	1	0.094108	0.923
SAPK/JNK Signaling	1.89	0.012882	0.094108	1	0.094108	0.923
IL-7 Signaling Pathway	1.89	0.012882	0.094108	1	0.094108	0.923
Apelin Endothelial Signaling Pathway	1.89	0.012882	0.094108	1	0.094108	0.923
Xenobiotic Metabolism Signaling	1.81	0.015488	0.111257	1	0.111257	0.815
FcγRIIB Signaling in B Lymphocytes	1.76	0.017378	0.11523	1	0.11523	1
IL-9 Signaling	1.76	0.017378	0.11523	1	0.11523	1
Virus Entry via Endocytic Pathways	1.76	0.017378	0.11523	1	0.11523	1
Docosahexaenoic Acid (DHA) Signaling	1.76	0.017378	0.11523	1	0.11523	1
Paxillin Signaling	1.76	0.017378	0.11523	1	0.11523	1
Erythropoietin Signaling	1.73	0.018621	0.116313	1	0.116313	0.875
CXCR4 Signaling	1.73	0.018621	0.116313	1	0.116313	0.875
Human Embryonic Stem Cell Pluripotency	1.73	0.018621	0.116313	1	0.116313	0.875
RANK Signaling in Osteoclasts	1.73	0.018621	0.116313	1	0.116313	0.875
Hereditary Breast Cancer Signaling	1.69	0.020417	0.120546	1	0.120546	0.917

HER-2 Signaling in Breast Cancer	1.69	0.020417	0.120546	1	0.120546	0.917
EGF Signaling	1.69	0.020417	0.120546	1	0.120546	0.917
Apoptosis Signaling	1.69	0.020417	0.120546	1	0.120546	0.917
GNRH Signaling	1.63	0.023442	0.134715	1	0.134715	0.842
Mouse Embryonic Stem Cell Pluripotency	1.63	0.023442	0.134715	1	0.134715	0.842
PTEN Signaling	1.57	0.026915	0.137311	1	0.137311	0.818
Wnt/β-catenin Signaling	1.57	0.026915	0.137311	1	0.137311	0.818
Melanocyte Development and Pigmentation Signaling	1.55	0.028184	0.137311	1	0.137311	0.867
Type I Diabetes Mellitus Signaling	1.55	0.028184	0.137311	1	0.137311	0.867
Acute Myeloid Leukemia Signaling	1.55	0.028184	0.137311	1	0.137311	0.867
Type II Diabetes Mellitus Signaling	1.55	0.028184	0.137311	1	0.137311	0.867
Breast Cancer Regulation by Stathmin1	1.55	0.028184	0.137311	1	0.137311	0.867
ErbB Signaling	1.55	0.028184	0.137311	1	0.137311	0.867
Tec Kinase Signaling	1.55	0.028184	0.137311	1	0.137311	0.867
Adipogenesis pathway	1.55	0.028184	0.137311	1	0.137311	0.867
IL-10 Signaling	1.54	0.02884	0.137311	1	0.137311	1
Lymphotoxin β Receptor Signaling	1.54	0.02884	0.137311	1	0.137311	1
Retinoic acid Mediated Apoptosis Signaling	1.54	0.02884	0.137311	1	0.137311	1
Apelin Pancreas Signaling Pathway	1.54	0.02884	0.137311	1	0.137311	1
Thrombopoietin Signaling	1.51	0.030903	0.137311	1	0.137311	0.909
Melanoma Signaling	1.51	0.030903	0.137311	1	0.137311	0.909
IL-17A Signaling in Airway Cells	1.51	0.030903	0.137311	1	0.137311	0.909
GDNF Family Ligand-Receptor Interactions	1.51	0.030903	0.137311	1	0.137311	0.909
UVC-Induced MAPK Signaling	1.51	0.030903	0.137311	1	0.137311	0.909
IL-2 Signaling	1.51	0.030903	0.137311	1	0.137311	0.909
Death Receptor Signaling	1.51	0.030903	0.137311	1	0.137311	0.909
SPINK1 General Cancer Pathway	1.51	0.030903	0.137311	1	0.137311	0.909
IL-8 Signaling	1.5	0.031623	0.137671	1	0.137671	0.786
Role of Osteoblasts, Osteoclasts and Chondrocytes in Rheumatoid Arthritis	1.5	0.031623	0.137671	1	0.137671	0.786
Neurotrophin/TRK Signaling	1.47	0.033884	0.146042	1	0.146042	0.833
Growth Hormone Signaling	1.39	0.040738	0.165642	1	0.165642	0.857
P2Y Purigenic Receptor Signaling Pathway	1.39	0.040738	0.165642	1	0.165642	0.857
Superpathway of Inositol Phosphate Compounds	1.39	0.040738	0.165642	1	0.165642	0.857
3-phosphoinositide Biosynthesis	1.39	0.040738	0.165642	1	0.165642	0.857
VEGF Signaling	1.39	0.040738	0.165642	1	0.165642	0.857
T Cell Receptor Signaling	1.39	0.040738	0.165642	1	0.165642	0.857

STAT3 Pathway	1.37	0.042658	0.16909	1	0.16909	0.778
Clathrin-mediated Endocytosis Signaling	1.32	0.047863	0.16909	1	0.16909	0.824
Bladder Cancer Signaling	1.32	0.047863	0.16909	1	0.16909	0.824
Role of NFAT in Cardiac Hypertrophy	1.32	0.047863	0.16909	1	0.16909	0.824
Integrin Signaling	1.32	0.047863	0.16909	1	0.16909	0.824
Amyotrophic Lateral Sclerosis Signaling	1.32	0.047863	0.16909	1	0.16909	0.9
CNTF Signaling	1.32	0.047863	0.16909	1	0.16909	0.9
Cdc42 Signaling	1.32	0.047863	0.16909	1	0.16909	0.9
Glioma Invasiveness Signaling	1.32	0.047863	0.16909	1	0.16909	0.9
ErbB4 Signaling	1.32	0.047863	0.16909	1	0.16909	0.9
GM-CSF Signaling	1.32	0.047863	0.16909	1	0.16909	0.9
Agrin Interactions at Neuromuscular Junction	1.32	0.047863	0.16909	1	0.16909	1
Sphingosine-1-phosphate Signaling	1.32	0.047863	0.16909	1	0.16909	1
Role of p14/p19ARF in Tumor Suppression	1.32	0.047863	0.16909	1	0.16909	1
Wnt/Ca+ pathway	1.32	0.047863	0.16909	1	0.16909	1
Cell Cycle: G1/S Checkpoint Regulation	1.32	0.047863	0.16909	1	0.16909	1
Relaxin Signaling	1.22	0.060256	0.199772	1	0.199772	0.846
FLT3 Signaling in Hematopoietic Progenitor Cells	1.22	0.060256	0.199772	1	0.199772	0.846
Aldosterone Signaling in Epithelial Cells	1.22	0.060256	0.199772	1	0.199772	0.846
ERK5 Signaling	1.22	0.060256	0.199772	1	0.199772	0.846
FAK Signaling	1.22	0.060256	0.199772	1	0.199772	0.846
PAK Signaling	1.22	0.060256	0.199772	1	0.199772	0.846
Rac Signaling	1.22	0.060256	0.199772	1	0.199772	0.846
Role of IL-17A in Arthritis	1.22	0.060256	0.199772	1	0.199772	0.846
Leukocyte Extravasation Signaling	1.17	0.067608	0.212695	1	0.212695	0.812
IL-12 Signaling and Production in Macrophages	1.17	0.067608	0.212695	1	0.212695	0.812
Renal Cell Carcinoma Signaling	1.17	0.067608	0.212695	1	0.212695	0.812
p70S6K Signaling	1.17	0.067608	0.212695	1	0.212695	0.812
Systemic Lupus Erythematosus Signaling	1.17	0.067608	0.212695	1	0.212695	0.812
PKCθ Signaling in T Lymphocytes	1.17	0.067608	0.212695	1	0.212695	0.812
JAK/Stat Signaling	1.17	0.067608	0.212695	1	0.212695	0.812
Role of NANOG in Mammalian Embryonic Stem Cell Pluripotency	1.14	0.072444	0.215882	1	0.215882	0.789
Induction of Apoptosis by HIV1	1.14	0.072444	0.215882	1	0.215882	0.889
Angiopoietin Signaling	1.14	0.072444	0.215882	1	0.215882	0.889
Regulation of eIF4 and p70S6K Signaling	1.14	0.072444	0.215882	1	0.215882	0.889

PCP pathway	1.14	0.072444	0.215882	1	0.215882	0.889
FGF Signaling	1.14	0.072444	0.215882	1	0.215882	0.889
Cancer Drug Resistance By Drug Efflux	1.14	0.072444	0.215882	1	0.215882	0.889
NRF2-mediated Oxidative Stress Response	1.13	0.074131	0.215882	1	0.215882	0.773
NF-κB Signaling	1.13	0.074131	0.215882	1	0.215882	0.773
Endocannabinoid Developing Neuron Pathway	1.13	0.074131	0.215882	1	0.215882	0.773
Endocannabinoid Cancer Inhibition Pathway	1.13	0.074131	0.215882	1	0.215882	0.773
Activation of IRF by Cytosolic Pattern Recognition Receptors	1.1	0.079433	0.218061	1	0.218061	1
TREM1 Signaling	1.1	0.079433	0.218061	1	0.218061	1
TNFR1 Signaling	1.1	0.079433	0.218061	1	0.218061	1
TNFR2 Signaling	1.1	0.079433	0.218061	1	0.218061	1
MSP-RON Signaling Pathway	1.1	0.079433	0.218061	1	0.218061	1
Inhibition of Angiogenesis by TSP1	1.1	0.079433	0.218061	1	0.218061	1
Cyclins and Cell Cycle Regulation	1.1	0.079433	0.218061	1	0.218061	1
Role of JAK family kinases in IL-6-type Cytokine Signaling	1.1	0.079433	0.218061	1	0.218061	1
iNOS Signaling	1.1	0.079433	0.218061	1	0.218061	1
Th1 Pathway	1.06	0.087096	0.237586	1	0.237586	0.833
Huntington's Disease Signaling	1	0.1	0.269375	1	0.269375	0.75
Adrenomedullin signaling pathway	1	0.1	0.269375	1	0.269375	0.75
Mitochondrial Dysfunction	0.959	0.109901	0.27863	1	0.27863	0.875
NF-κB Activation by Viruses	0.959	0.109901	0.27863	1	0.27863	0.875
Neuropathic Pain Signaling In Dorsal Horn Neurons	0.959	0.109901	0.27863	1	0.27863	0.875
G Beta Gamma Signaling	0.959	0.109901	0.27863	1	0.27863	0.875
Role of Wnt/GSK-3β Signaling in the Pathogenesis of Influenza	0.959	0.109901	0.27863	1	0.27863	0.875
RhoGDI Signaling	0.959	0.109901	0.27863	1	0.27863	0.875
Ephrin B Signaling	0.959	0.109901	0.27863	1	0.27863	0.875
Regulation of Cellular Mechanics by Calpain Protease	0.959	0.109901	0.27863	1	0.27863	0.875
Estrogen Receptor Signaling	0.959	0.109901	0.27863	1	0.27863	0.875
Hypoxia Signaling in the Cardiovascular System	0.959	0.109901	0.27863	1	0.27863	0.875
Glucocorticoid Receptor Signaling	0.936	0.115878	0.292066	1	0.292066	0.714
CREB Signaling in Neurons	0.91	0.123027	0.302998	1	0.302998	0.818
Role of JAK1 and JAK3 in γc Cytokine Signaling	0.91	0.123027	0.302998	1	0.302998	0.818
IL-4 Signaling	0.91	0.123027	0.302998	1	0.302998	0.818

Chemokine Signaling	0.91	0.123027	0.302998	1	0.302998	0.818
Acute Phase Response Signaling	0.9	0.125893	0.308293	1	0.308293	0.731
IL-3 Signaling	0.889	0.129122	0.308293	1	0.308293	0.786
Cholecystokinin/Gastrin-mediated Signaling	0.883	0.130918	0.308293	1	0.308293	0.765
Role of PKR in Interferon Induction and Antiviral Response	0.876	0.133045	0.308293	1	0.308293	1
B Cell Activating Factor Signaling	0.876	0.133045	0.308293	1	0.308293	1
April Mediated Signaling	0.876	0.133045	0.308293	1	0.308293	1
IL-17A Signaling in Fibroblasts	0.876	0.133045	0.308293	1	0.308293	1
GADD45 Signaling	0.876	0.133045	0.308293	1	0.308293	1
Fatty Acid α-oxidation	0.876	0.133045	0.308293	1	0.308293	1
Unfolded protein response	0.876	0.133045	0.308293	1	0.308293	1
Cell Cycle: G2/M DNA Damage Checkpoint Regulation	0.876	0.133045	0.308293	1	0.308293	1
4-1BB Signaling in T Lymphocytes	0.785	0.164059	0.370206	1	0.370206	0.857
Oncostatin M Signaling	0.785	0.164059	0.370206	1	0.370206	0.857
Role of IL-17F in Allergic Inflammatory Airway Diseases	0.785	0.164059	0.370206	1	0.370206	0.857
Toll-like Receptor Signaling	0.785	0.164059	0.370206	1	0.370206	0.857
GP6 Signaling Pathway	0.785	0.164059	0.370206	1	0.370206	0.857
Thrombin Signaling	0.762	0.172982	0.371519	1	0.371519	0.75
HMGB1 Signaling	0.762	0.172982	0.371519	1	0.371519	0.75
eNOS Signaling	0.762	0.172982	0.371519	1	0.371519	0.8
Neuregulin Signaling	0.757	0.174985	0.371519	1	0.371519	0.769
CD40 Signaling	0.757	0.174985	0.371519	1	0.371519	0.769
IL-15 Signaling	0.757	0.174985	0.371519	1	0.371519	0.769
Dendritic Cell Maturation	0.757	0.174985	0.371519	1	0.371519	0.769
Reelin Signaling in Neurons	0.757	0.174985	0.371519	1	0.371519	0.769
PI3K Signaling in B Lymphocytes	0.757	0.174985	0.371519	1	0.371519	0.769
Epithelial Adherens Junction Signaling	0.757	0.174985	0.371519	1	0.371519	0.769
TGF-β Signaling	0.757	0.174985	0.371519	1	0.371519	0.769
BMP signaling pathway	0.757	0.174985	0.371519	1	0.371519	0.769
Axonal Guidance Signaling	0.73	0.186209	0.393412	1	0.393412	0.683
Role of NFAT in Regulation of the Immune Response	0.662	0.217771	0.434543	1	0.434543	0.722
Signaling by Rho Family GTPases	0.662	0.217771	0.434543	1	0.434543	0.722
Role of BRCA1 in DNA Damage Response	0.656	0.2208	0.434543	1	0.434543	1
IL-22 Signaling	0.656	0.2208	0.434543	1	0.434543	1
Mitotic Roles of Polo-Like Kinase	0.656	0.2208	0.434543	1	0.434543	1
Actin Nucleation by ARP-WASP Complex	0.656	0.2208	0.434543	1	0.434543	1

Dopamine Degradation	0.656	0.2208	0.434543	1	0.434543	1
Retinol Biosynthesis	0.656	0.2208	0.434543	1	0.434543	1
Histamine Degradation	0.656	0.2208	0.434543	1	0.434543	1
Noradrenaline and Adrenaline Degradation	0.656	0.2208	0.434543	1	0.434543	1
Putrescine Degradation III	0.656	0.2208	0.434543	1	0.434543	1
Oxidative Ethanol Degradation III	0.656	0.2208	0.434543	1	0.434543	1
Ethanol Degradation IV	0.656	0.2208	0.434543	1	0.434543	1
Ethanol Degradation II	0.656	0.2208	0.434543	1	0.434543	1
Th17 Activation Pathway	0.656	0.2208	0.434543	1	0.434543	1
CD28 Signaling in T Helper Cells	0.646	0.225944	0.440641	1	0.440641	0.733
Th1 and Th2 Activation Pathway	0.646	0.225944	0.440641	1	0.440641	0.733
Gαq Signaling	0.631	0.233884	0.445645	1	0.445645	0.75
VDR/RXR Activation	0.62	0.239883	0.445645	1	0.445645	0.833
CD27 Signaling in Lymphocytes	0.62	0.239883	0.445645	1	0.445645	0.778
Androgen Signaling	0.62	0.239883	0.445645	1	0.445645	0.833
IL-1 Signaling	0.62	0.239883	0.445645	1	0.445645	0.833
Role of PI3K/AKT Signaling in the Pathogenesis of Influenza	0.62	0.239883	0.445645	1	0.445645	0.778
OX40 Signaling Pathway	0.62	0.239883	0.445645	1	0.445645	0.833
IL-17A Signaling in Gastric Cells	0.62	0.239883	0.445645	1	0.445645	0.833
Remodeling of Epithelial Adherens Junctions	0.62	0.239883	0.445645	1	0.445645	0.833
Macropinocytosis Signaling	0.62	0.239883	0.445645	1	0.445645	0.778
Apelin Cardiomyocyte Signaling Pathway	0.62	0.239883	0.445645	1	0.445645	0.833
Hepatic Cholestasis	0.561	0.274789	0.508301	1	0.508301	0.706
Th2 Pathway	0.538	0.289734	0.533656	1	0.533656	0.714
Hepatic Fibrosis / Hepatic Stellate Cell Activation	0.514	0.306196	0.558122	1	0.558122	0.667
Salvage Pathways of Pyrimidine Ribonucleotides	0.513	0.306902	0.558122	1	0.558122	0.727
Iron homeostasis signaling pathway	0.513	0.306902	0.558122	1	0.558122	0.727
CCR5 Signaling in Macrophages	0.488	0.325087	0.577184	1	0.577184	0.75
Factors Promoting Cardiogenesis in Vertebrates	0.488	0.325087	0.577184	1	0.577184	0.75
Crosstalk between Dendritic Cells and Natural Killer Cells	0.488	0.325087	0.577184	1	0.577184	0.75
Apelin Liver Signaling Pathway	0.488	0.325087	0.577184	1	0.577184	0.75
Gap Junction Signaling	0.466	0.341979	0.577184	1	0.577184	0.688
Caveolar-mediated Endocytosis Signaling	0.462	0.345144	0.577184	1	0.577184	0.8
Graft-versus-Host Disease Signaling	0.462	0.345144	0.577184	1	0.577184	0.8

Role of Hypercytokinemia/hyperchemokinemia in the Pathogenesis of Influenza	0.462	0.345144	0.577184	1	0.577184	0.8
Ephrin A Signaling	0.462	0.345144	0.577184	1	0.577184	0.8
D-myo-inositol-5-phosphate Metabolism	0.462	0.345144	0.577184	1	0.577184	0.8
D-myo-inositol (1,4,5,6)-Tetrakisphosphate Biosynthesis	0.462	0.345144	0.577184	1	0.577184	0.8
D-myo-inositol (3,4,5,6)-tetrakisphosphate Biosynthesis	0.462	0.345144	0.577184	1	0.577184	0.8
3-phosphoinositide Degradation	0.462	0.345144	0.577184	1	0.577184	0.8
Amyloid Processing	0.462	0.345144	0.577184	1	0.577184	0.8
ERK/MAPK Signaling	0.444	0.359749	0.577184	1	0.577184	0.655
Renin-Angiotensin Signaling	0.438	0.364754	0.577184	1	0.577184	0.667
Fcγ Receptor-mediated Phagocytosis in Macrophages and Monocytes	0.437	0.365595	0.577184	1	0.577184	1
Cytotoxic T Lymphocyte-mediated Apoptosis of Target Cells	0.437	0.365595	0.577184	1	0.577184	1
IL-15 Production	0.437	0.365595	0.577184	1	0.577184	1
Polyamine Regulation in Colon Cancer	0.437	0.365595	0.577184	1	0.577184	1
Extrinsic Prothrombin Activation Pathway	0.437	0.365595	0.577184	1	0.577184	1
Tumoricidal Function of Hepatic Natural Killer Cells	0.437	0.365595	0.577184	1	0.577184	1
DNA Double-Strand Break Repair by Homologous Recombination	0.437	0.365595	0.577184	1	0.577184	1
Transcriptional Regulatory Network in Embryonic Stem Cells	0.437	0.365595	0.577184	1	0.577184	1
Estrogen-mediated S-phase Entry	0.437	0.365595	0.577184	1	0.577184	1
Heparan Sulfate Biosynthesis	0.437	0.365595	0.577184	1	0.577184	1
Heparan Sulfate Biosynthesis (Late Stages)	0.437	0.365595	0.577184	1	0.577184	1
Triacylglycerol Degradation	0.437	0.365595	0.577184	1	0.577184	1
Triacylglycerol Biosynthesis	0.437	0.365595	0.577184	1	0.577184	1
Superpathway of Geranylgeranyldiphosphate Biosynthesis I (via Mevalonate)	0.437	0.365595	0.577184	1	0.577184	1
Superpathway of Cholesterol Biosynthesis	0.437	0.365595	0.577184	1	0.577184	1
HIPPO signaling	0.437	0.365595	0.577184	1	0.577184	1
Interferon Signaling	0.437	0.365595	0.577184	1	0.577184	1
Endoplasmic Reticulum Stress Pathway	0.437	0.365595	0.577184	1	0.577184	1
Notch Signaling	0.437	0.365595	0.577184	1	0.577184	1

SPINK1 Pancreatic Cancer Pathway	0.437	0.365595	0.577184	1	0.577184	1
Neuroinflammation Signaling Pathway	0.416	0.383707	0.603569	1	0.603569	0.641
LPS/IL-1 Mediated Inhibition of RXR Function	0.41	0.389045	0.607957	1	0.607957	0.667
Natural Killer Cell Signaling	0.405	0.39355	0.607957	1	0.607957	0.7
CDK5 Signaling	0.405	0.39355	0.607957	1	0.607957	0.7
Leptin Signaling in Obesity	0.405	0.39355	0.607957	1	0.607957	0.7
Pyridoxal 5'-phosphate Salvage Pathway	0.405	0.39355	0.607957	1	0.607957	0.7
Actin Cytoskeleton Signaling	0.379	0.41783	0.64316	1	0.64316	0.667
Synaptic Long Term Potentiation	0.366	0.430527	0.655678	1	0.655678	0.714
CTLA4 Signaling in Cytotoxic T Lymphocytes	0.366	0.430527	0.655678	1	0.655678	0.714
Role of Oct4 in Mammalian Embryonic Stem Cell Pluripotency	0.366	0.430527	0.655678	1	0.655678	0.714
G-Protein Coupled Receptor Signaling	0.353	0.443609	0.673223	1	0.673223	0.64
fMLP Signaling in Neutrophils	0.345	0.451856	0.678571	1	0.678571	0.667
Regulation of IL-2 Expression in Activated and Anergic T Lymphocytes	0.345	0.451856	0.678571	1	0.678571	0.667
Nitric Oxide Signaling in the Cardiovascular System	0.345	0.451856	0.678571	1	0.678571	0.667
Airway Pathology in Chronic Obstructive Pulmonary Disease	0.315	0.484172	0.704994	1	0.704994	0.75
Role of CHK Proteins in Cell Cycle Checkpoint Control	0.315	0.484172	0.704994	1	0.704994	0.75
Cell Cycle Control of Chromosomal Replication	0.315	0.484172	0.704994	1	0.704994	0.75
Differential Regulation of Cytokine Production in Macrophages and T Helper Cells by IL-17A and IL-17F	0.315	0.484172	0.704994	1	0.704994	0.75
Differential Regulation of Cytokine Production in Intestinal Epithelial Cells by IL-17A and IL-17F	0.315	0.484172	0.704994	1	0.704994	0.75
Tryptophan Degradation X (Mammalian, via Tryptamine)	0.315	0.484172	0.704994	1	0.704994	0.75
Serotonin Degradation	0.315	0.484172	0.704994	1	0.704994	0.75
Inhibition of Matrix Metalloproteases	0.315	0.484172	0.704994	1	0.704994	0.75
Cardiac β-adrenergic Signaling	0.315	0.484172	0.704994	1	0.704994	0.75
Tight Junction Signaling	0.305	0.49545	0.711797	1	0.711797	0.667
Role of Pattern Recognition Receptors in Recognition of Bacteria and Viruses	0.305	0.49545	0.711797	1	0.711797	0.667
iCOS-iCOSL Signaling in T Helper Cells	0.305	0.49545	0.711797	1	0.711797	0.667
Communication between Innate and	0.305	0.49545	0.711797	1	0.711797	0.667

Adaptive Immune Cells						
PXR/RXR Activation	0.262	0.547016	0.730456	1	0.730456	0.636
Basal Cell Carcinoma Signaling	0.262	0.547016	0.730456	1	0.730456	0.636
α-Adrenergic Signaling	0.256	0.554626	0.730456	1	0.730456	0.667
Allograft Rejection Signaling	0.256	0.554626	0.730456	1	0.730456	0.667
Altered T Cell and B Cell Signaling in Rheumatoid Arthritis	0.256	0.554626	0.730456	1	0.730456	0.667
Phagosome Formation	0.256	0.554626	0.730456	1	0.730456	0.667
Opioid Signaling Pathway	0.238	0.578096	0.730456	1	0.730456	0.609
LXR/RXR Activation	0.234	0.583445	0.730456	1	0.730456	0.611
Antiproliferative Role of Somatostatin Receptor 2	0.228	0.591562	0.730456	1	0.730456	0.615
Role of RIG1-like Receptors in Antiviral Innate Immunity	0.218	0.605341	0.730456	1	0.730456	1
Mechanisms of Viral Exit from Host Cells	0.218	0.605341	0.730456	1	0.730456	1
Cell Cycle Regulation by BTG Family Proteins	0.218	0.605341	0.730456	1	0.730456	1
Maturity Onset Diabetes of Young (MODY) Signaling	0.218	0.605341	0.730456	1	0.730456	1
Granzyme A Signaling	0.218	0.605341	0.730456	1	0.730456	1
TWEAK Signaling	0.218	0.605341	0.730456	1	0.730456	1
Role of Lipids/Lipid Rafts in the Pathogenesis of Influenza	0.218	0.605341	0.730456	1	0.730456	1
Role of IL-17A in Psoriasis	0.218	0.605341	0.730456	1	0.730456	1
Role of JAK1, JAK2 and TYK2 in Interferon Signaling	0.218	0.605341	0.730456	1	0.730456	1
NAD Biosynthesis from 2-amino-3-carboxymuconate Semialdehyde	0.218	0.605341	0.730456	1	0.730456	1
NAD biosynthesis II (from tryptophan)	0.218	0.605341	0.730456	1	0.730456	1
Arginine Biosynthesis IV	0.218	0.605341	0.730456	1	0.730456	1
Retinoate Biosynthesis II	0.218	0.605341	0.730456	1	0.730456	1
Proline Biosynthesis II (from Arginine)	0.218	0.605341	0.730456	1	0.730456	1
Glutathione Redox Reactions I	0.218	0.605341	0.730456	1	0.730456	1
Heme Degradation	0.218	0.605341	0.730456	1	0.730456	1
Tetrahydrofolate Salvage from 5,10-methenyltetrahydrofolate	0.218	0.605341	0.730456	1	0.730456	1
Ceramide Biosynthesis	0.218	0.605341	0.730456	1	0.730456	1
γ-linolenate Biosynthesis II (Animals)	0.218	0.605341	0.730456	1	0.730456	1
Guanine and Guanosine Salvage I	0.218	0.605341	0.730456	1	0.730456	1
Oleate Biosynthesis II (Animals)	0.218	0.605341	0.730456	1	0.730456	1
Arginine Degradation VI (Arginase 2 Pathway)	0.218	0.605341	0.730456	1	0.730456	1

5-aminoimidazole Ribonucleotide Biosynthesis I	0.218	0.605341	0.730456	1	0.730456	1
S-methyl-5'-thioadenosine Degradation II	0.218	0.605341	0.730456	1	0.730456	1
Glycogen Degradation III	0.218	0.605341	0.730456	1	0.730456	1
Calcium Transport I	0.218	0.605341	0.730456	1	0.730456	1
Glycogen Degradation II	0.218	0.605341	0.730456	1	0.730456	1
Citrulline Biosynthesis	0.218	0.605341	0.730456	1	0.730456	1
Mevalonate Pathway I	0.218	0.605341	0.730456	1	0.730456	1
Palmitate Biosynthesis I (Animals)	0.218	0.605341	0.730456	1	0.730456	1
Glutathione Redox Reactions II	0.218	0.605341	0.730456	1	0.730456	1
Purine Nucleotides De Novo Biosynthesis II	0.218	0.605341	0.730456	1	0.730456	1
Methylglyoxal Degradation III	0.218	0.605341	0.730456	1	0.730456	1
Fatty Acid Biosynthesis Initiation II	0.218	0.605341	0.730456	1	0.730456	1
Stearate Biosynthesis I (Animals)	0.218	0.605341	0.730456	1	0.730456	1
Arginine Degradation I (Arginase Pathway)	0.218	0.605341	0.730456	1	0.730456	1
Trans, trans-farnesyl Diphosphate Biosynthesis	0.218	0.605341	0.730456	1	0.730456	1
Methionine Degradation I (to Homocysteine)	0.218	0.605341	0.730456	1	0.730456	1
Superpathway of Methionine Degradation	0.218	0.605341	0.730456	1	0.730456	1
Molybdenum Cofactor Biosynthesis	0.218	0.605341	0.730456	1	0.730456	1
Folate Polyglutamylation	0.218	0.605341	0.730456	1	0.730456	1
S-adenosyl-L-methionine Biosynthesis	0.218	0.605341	0.730456	1	0.730456	1
Cysteine Biosynthesis III (mammalia)	0.218	0.605341	0.730456	1	0.730456	1
Vitamin-C Transport	0.218	0.605341	0.730456	1	0.730456	1
Oxidative Phosphorylation	0.218	0.605341	0.730456	1	0.730456	1
BER pathway	0.218	0.605341	0.730456	1	0.730456	1
Antigen Presentation Pathway	0.218	0.605341	0.730456	1	0.730456	1
RhoA Signaling	0.217	0.606736	0.730456	1	0.730456	0.625
Role of JAK2 in Hormone-like Cytokine Signaling	0.217	0.606736	0.730456	1	0.730456	0.625
Role of Cytokines in Mediating Communication between Immune Cells	0.183	0.656145	0.772674	1	0.772674	0.667
Semaphorin Signaling in Neurons	0.183	0.656145	0.772674	1	0.772674	0.667
G Protein Signaling Mediated by Tubby	0.183	0.656145	0.772674	1	0.772674	0.667
Pathogenesis of Multiple Sclerosis	0.183	0.656145	0.772674	1	0.772674	0.667
Retinoate Biosynthesis I	0.183	0.656145	0.772674	1	0.772674	0.667
DNA damage-induced 14-3-3 σ Signaling	0.183	0.656145	0.772674	1	0.772674	0.667

GABA Receptor Signaling	0.183	0.656145	0.772674	1	0.772674	0.667
autophagy	0.183	0.656145	0.772674	1	0.772674	0.667
Circadian Rhythm Signaling	0	1	1	1	1	0.5
Fc Epsilon RI Signaling	0	1	1	1	1	0.476
Synaptic Long Term Depression	0	1	1	1	1	0.294
Coagulation System	0	1	1	1	1	0.571
FXR/RXR Activation	0	1	1	1	1	0.562
Regulation of Actin-based Motility by Rho	0	1	1	1	1	0.5
MIF Regulation of Innate Immunity	0	1	1	1	1	0.429
Airway Inflammation in Asthma	0	1	1	1	1	0.5
T Helper Cell Differentiation	0	1	1	1	1	0.429
CCR3 Signaling in Eosinophils	0	1	1	1	1	0.421
Melatonin Signaling	0	1	1	1	1	0.571
Cellular Effects of Sildenafil (Viagra)	0	1	1	1	1	0.2
Endothelin-1 Signaling	0	1	1	1	1	0.517
Cardiomyocyte Differentiation via BMP Receptors	0	1	1	1	1	0.5
Corticotropin Releasing Hormone Signaling	0	1	1	1	1	0.5
Autoimmune Thyroid Disease Signaling	0	1	1	1	1	0.571
Phospholipase C Signaling	0	1	1	1	1	0.429
Atherosclerosis Signaling	0	1	1	1	1	0.438
Protein Kinase A Signaling	0	1	1	1	1	0.591
B Cell Development	0	1	1	1	1	0.5
Neuroprotective Role of THOP1 in Alzheimer's Disease	0	1	1	1	1	0.364
Nur77 Signaling in T Lymphocytes	0	1	1	1	1	0.571
Role of MAPK Signaling in the Pathogenesis of Influenza	0	1	1	1	1	0.474
MIF-mediated Glucocorticoid Regulation	0	1	1	1	1	0.273
DNA Double-Strand Break Repair by Non-Homologous End Joining	0	1	1	1	1	0.5
Dopamine-DARPP32 Feedback in cAMP Signaling	0	1	1	1	1	0.333
Telomere Extension by Telomerase	0	1	1	1	1	0.5
Hematopoiesis from Multipotent Stem Cells	0	1	1	1	1	0.5
nNOS Signaling in Skeletal Muscle Cells	0	1	1	1	1	0.333
VEGF Family Ligand-Receptor Interactions	0	1	1	1	1	0.524
Netrin Signaling	0	1	1	1	1	0.5

Bupropion Degradation	0	1	1	1	1	0.4
Nicotine Degradation III	0	1	1	1	1	0.333
Prostanoid Biosynthesis	0	1	1	1	1	0.5
Superpathway of Citrulline Metabolism	0	1	1	1	1	0.5
The Visual Cycle	0	1	1	1	1	0.5
Melatonin Degradation I	0	1	1	1	1	0.333
Estrogen Biosynthesis	0	1	1	1	1	0.4
Nicotine Degradation II	0	1	1	1	1	0.333
Superoxide Radicals Degradation	0	1	1	1	1	0.5
Adenine and Adenosine Salvage III	0	1	1	1	1	0.333
Superpathway of Melatonin Degradation	0	1	1	1	1	0.333
Acetone Degradation I (to Methylglyoxal)	0	1	1	1	1	0.4
Antioxidant Action of Vitamin C	0	1	1	1	1	0.333
Gαi Signaling	0	1	1	1	1	0.545
Gαs Signaling	0	1	1	1	1	0.429
Agranulocyte Adhesion and Diapedesis	0	1	1	1	1	0.533
Granulocyte Adhesion and Diapedesis	0	1	1	1	1	0.533
Sperm Motility	0	1	1	1	1	0.182
Protein Ubiquitination Pathway	0	1	1	1	1	0.6
Phototransduction Pathway	0	1	1	1	1	0.5
Sonic Hedgehog Signaling	0	1	1	1	1	0.5
Dopamine Receptor Signaling	0	1	1	1	1	0.333
cAMP-mediated signaling	0	1	1	1	1	0.526
p38 MAPK Signaling	0	1	1	1	1	0.556
Glutamate Receptor Signaling	0	1	1	1	1	0.333
GPCR-Mediated Integration of Enteroendocrine Signaling Exemplified by an L Cell	0	1	1	1	1	0.333
GPCR-Mediated Nutrient Sensing in Enteroendocrine Cells	0	1	1	1	1	0.25
Gustation Pathway	0	1	1	1	1	0.5
Phagosome Maturation	0	1	1	1	1	0.333
Apelin Cardiac Fibroblast Signaling Pathway	0	1	1	1	1	0.5
Apelin Adipocyte Signaling Pathway	0	1	1	1	1	0.6
Calcium Signaling	0	1	1	1	1	0.444
Complement System	0	1	1	1	1	0.6
Eicosanoid Signaling	0	1	1	1	1	0.1
